# A Sustainable Green Enzymatic Method for Amide Bond Formation

**DOI:** 10.3390/molecules28155706

**Published:** 2023-07-28

**Authors:** György Orsy, Sayeh Shahmohammadi, Enikő Forró

**Affiliations:** 1 Institute of Pharmaceutical Chemistry, University of Szeged, Eötvös u. 6, H-6720 Szeged, Hungary; orsy.gyorgy@szte.hu (G.O.); sayeh.s@pharm.u-szeged.hu (S.S.); 2 Stereochemistry Research Group, Eötvös Loránd Research Network, University of Szeged, Eötvös u. 6, H-6720 Szeged, Hungary

**Keywords:** sustainable enzymatic strategy, direct amide synthesis, green solvent, CALB, carboxylic acid, amine

## Abstract

A sustainable enzymatic strategy for the preparation of amides by using *Candida antarctica* lipase B as the biocatalyst and cyclopentyl methyl ether as a green and safe solvent was devised. The method is simple and efficient and it produces amides with excellent conversions and yields without the need for intensive purification steps. The scope of the reaction was extended to the preparation of 28 diverse amides using four different free carboxylic acids and seven primary and secondary amines, including cyclic amines. This enzymatic methodology has the potential to become a green and industrially reliable process for direct amide synthesis.

## 1. Introduction

The amide bond is a fundamental linkage in nature. It is the main chemical bond that links amino acid building blocks together to give peptides and proteins, which occur worldwide [1,2,3]. Furthermore, as an important moiety of pharmaceutically active compounds, it can be found in a significant array of commercial drugs worldwide. For example, Acetaminophen, a common pain reliever and an antipyretic agent, is used to treat various conditions such as headache, muscle aches, and arthritis [4]. Amide-based local anesthetics are applied to numb a specific area of the body, before a medical procedure or surgery [5,6]. β-Lactam antibiotics are a group of antibiotics that are used to treat bacterial infections, including pneumonia, bronchitis, and urinary tract infections [7,8,9]. Celecoxib is a nonsteroidal anti-inflammatory drug (NSAID) utilized to treat pain and inflammation associated with conditions, such as arthritis, menstrual cramps, and sport injuries [10,11]. These are just a few examples of amide drugs, and there are many others used in the treatment of various medical conditions.

A large number of synthetic methods resulting in the formation of amide bonds have been devised in the last decade [12,13,14,15,16,17,18,19]. However, there are only a limited number of strategies that are both efficient and environmentally benign [20,21,22]. The most common processes utilize coupling reagents or activating agents with larger stoichiometric ratio to couple a free carboxylic acid with an amine. However, these are generally hazardous/poisonous reagents and, consequently, they put a heavy burden on the environment. Furthermore, the purification of the crude products is problematic, since it requires a large quantity of organic solvents, due to the formation of large quantities of by-products [23,24]. Therefore, there is a great demand to develop simple amide-bond-forming reactions to access amides from free carboxylic acids and amines in a green and efficient way.

The green chemistry concept has 12 principles aimed at the design of chemical products and processes, that reduce or eliminate the application and generation of hazardous substances, which are harmful for human health or the environment [25,26]. Using enzymes in synthetic chemistry has always been a hot topic due to the ability of enzymes to catalyze chemical transformations with high catalytic efficiency and specificity [27,28,29,30,31]. For instance, the reactions mentioned above carried out under harsh conditions can be induced to proceed faster under mild conditions (lower temperatures and pressures, neutral pH), with fewer work-up steps and higher yields. All of these result in an improvement in efficiency and save energy. Enzymes have emerged as preferred tools in green chemistry by replacing hazardous/poisonous reagents, generating the formation of fewer by-products [32,33,34,35].

In the case of amide bond formation, enzymes, particularly the members of the lipase family, are powerful and effective biocatalysts for esterification reactions [36,37,38]. Several lipases have been reported to exhibit high catalytic activity and stability in organic solvents [39,40]. In particular, *Candida antarctica* lipase B (CALB; Novozyme 435) prefers anhydrous conditions and it has been widely applied in esterification and hydrolysis studies [41,42,43,44,45,46]. CALB could also catalyze amidation reactions [47,48,49,50,51,52] when the amine is used as a nucleophile in anhydrous organic media. In these reactions, amine amidation with a free carboxylic acid takes place, resulting in the amide product. A previous study reported by the Manova group [53] showed that CALB could be a simple and convenient biocatalyst for the efficient, direct amidation of free carboxylic acids with amines applied for a wide range of substrates, including lipoic acid.

Thus, the CALB enzymatic approach could offer the possibility of accomplishing direct amide coupling in an efficient and sustainable way without any additives in green organic solvents, providing amides with high yields and excellent purity.

**Scheme 1:**
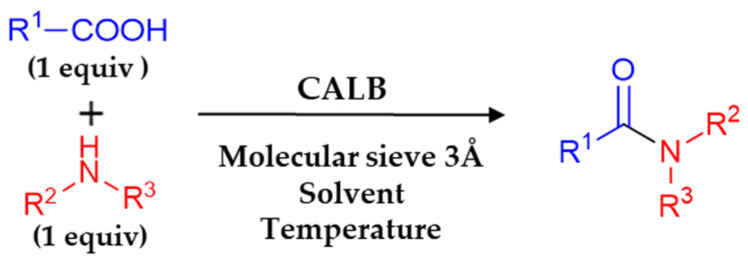
CALB-catalyzed synthesis of amides.

Herein, we report a sustainable amidation strategy through CALB-catalyzed coupling of free acyclic carboxylic acids with different primary and secondary amines in a prominent green solvent (Scheme 1). In order to follow the progress of the enzymatic reactions, we also develop an adequate gas chromatography–mass spectrometry (GC-MS) analytic method.

## 2. Results and Discussion

**Figure 1:**
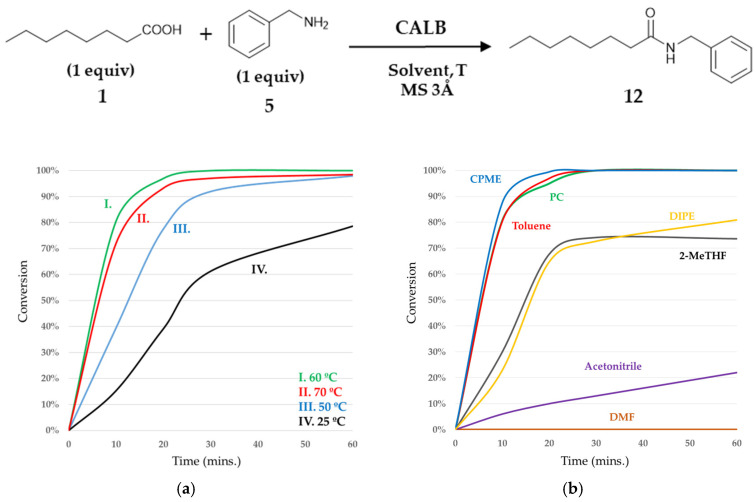
The effect of temperature (**a**) and solvents *(***b***)* on the reaction conversion catalyzed by CALB.

In view of the results on the enzymatic amidation of free carboxylic acids with amines [49], CALB-catalyzed amidation of octanoic acid (**1**) with benzylamine (**5**) in the presence of a molecular sieve in toluene at 60 °C was performed (Figure 1a curve I). An excellent conversion of >99% after 30 min was observed.

In order to increase the reaction rate, further preliminary experiments were performed at different temperatures (Figure 1a curves II–IV). When the amidation was performed at 25 °C, the desired product with 78% conversion was obtained after 30 min (curve IV). By applying a higher temperature of 50 °C, the conversion of the reaction improved remarkably, reaching a >99% conversion in 60 min (curve III). However, at temperatures higher than 60 °C, the conversion decreased slightly, because of the thermal denaturation process of CALB protein chains (curve II). The optimal temperature of 60 °C was chosen for further reactions.

Next, we screened organic solvents with different types of polarity such as acetonitrile and N,N-dimethylformamide (DMF). In addition, we focused on the application of greener alternative solvents, such as propylene carbonate (PC), 2-methyltetrahydrofuran (2-MeTHF), diisopropyl ether (DIPE), and cyclopentyl methyl ether (CPME). While the reaction rate in acetonitrile was relatively low, CPME and PC were the most promising green solvents with conversions reaching >99% in 30 min (Figure 1b).

**Figure 2:**
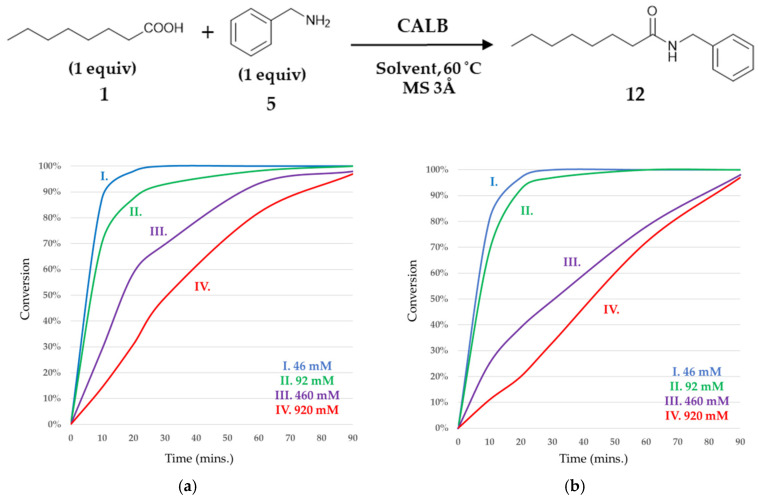
The efficiency of amide formation catalyzed by CALB in CPME (**a**) and PC (**b**).

In an attempt to increase the efficiency of the present method, the initial concentration of the substrate of 46 mM was increased to 92, 460, and 920 mM (Figure 2a,b). Reactions were performed in CPME and PC solvents with a constant enzyme concentration of 50 mg mL^–1^. Slightly lower reaction rates with 92 mM concentration (curves II(**a**) and (**b**)) were observed. Despite the much lower reaction rates found at concentrations of 460 and 920 mM, the amide formation was still significant after 60 min (curves III(**a**) and (**b**) and IV(**a**) and (**b**)). Such robust behavior of commercially available CALB might be an important parameter not only for laboratory-scale but also industrial-scale reactions [54,55].

Having in hand the optimal conditions (CALB, molecular sieve 3 Å, CPME solvent, 60 °C, substrate concentration 920 mM), we performed further amidation reactions and obtained 28 different amides (**12**–**39**) with excellent conversions (>92%) and yields (>90%) in 90 min (Table 1). The study involved the use of four different free carboxylic acids and seven amines including primary, secondary, and secondary cyclic amines. According to GC-MS analysis, all reactions were completed in 90 min. The product molecules might be used as potential intermediates or building blocks in the synthesis of biologically active compounds [56,57].

## 3. Materials and Methods

### 3.1. General

All solvents and reagents were of analytical grade and used directly without further purification. The lipase acrylic resin (CALB, ≥5000 U/g, recombinant, expressed in Aspergillus niger, quality level 200, Catalogue no. L4777), cyclopentyl methyl ether (inhibitor-free, anhydrous, ≥99.9%), propylene carbonate (ReagentPlus^®^, 99%), toluene (anhydrous, 99.8%), 2-methyltetrahydrofuran (BioRenewable, anhydrous, ≥99%, inhibitor-free), acetonitrile (suitable for HPLC, gradient grade, ≥99.9%), *N,N*-dimethylformamide (suitable for HPLC, ≥99.9%), and 3 Å molecular sieve (beads, 8–12 mesh) used in this study were purchased from Merck Life Science Kft., an affiliate of Merck KGaA, Darmstadt, Germany (Budapest, Hungary). All reactions were carried out in an Eppendorf™ Innova™ 40R Incubator Shaker. Melting points were determined on a Kofler apparatus. ^1^H NMR and ^13^C NMR spectra were recorded on a Bruker Avance NEO 500.1 spectrometer, in CDCl_3_ as solvent, with tetramethylsilane as an internal standard at 500.1 and 125 MHz, respectively. GC-MS analyses were performed on a Thermo Scientific Trace 1310 Gas Chromatograph coupled with a Thermo Scientific ISQ QD Single-Quadrupole Mass Spectrometer using a Thermo Scientific TG-SQC column (15 m × 0.25 mm ID × 0.25 μm film). Measurement parameters were as follows: column oven temperature: from 50 to 300 °C at 15 °C min^−1^; injection temperature: 240 °C; ion source temperature: 200 °C; electrospray ionization: 70 eV; carrier gas: He at 1.5 mL min^−1^; injection volume: 5 μL; split ratio: 1:50; and mass range: 25–500 m/z.

### 3.2. General Procedure for CALB-Catalyzed Amidation

In preliminary amidation experiments, the carboxylic acid and amine substrates (1:1 equiv) were dissolved in an organic solvent (1 mL) to provide a solution with a given concentration (46, 92, 460, or 920 mM). CALB (50 mg), molecular sieve (50 mg 3 Å size) to avoid the reversible hydrolysis reaction, and *n*-heptadecane (2 µL) as an internal standard were added to the above solution. The mixture was shaken at a selected temperature (25, 50, 60 or 70 °C) in an incubator shaker. The progress of the reaction was followed by taking samples from the reaction mixture at intervals and analyzing them by GC-MS measurements. Conversion of the starting materials (moles of the converted molecules/moles of the initial starting materials × 100) was calculated by *n*-heptadecane as an internal standard, in percent yield of the desired amides (actual yield/theoretical yield × 100) (see Supplementary Information). In order to obtain reliable yields, we filtered the CPME samples through a silica gel plug followed by vacuum evaporation of the solvent. All samples were analyzed by ^1^H and ^13^C NMR spectroscopy without any prior purification. Amidations, to form amides **12**–**39**, were performed with the above protocol under the optimized conditions (50 mg CALB, 920 mM substrate concentration, 1 mL solvent CPME, 50 mg molecular sieve, 2 µL *n*-heptadecane, 60 °C).

#### 3.2.1. N-Benzyloctanamide (**12**)

CAS number: 70659-87-9, white solid, mp = 65.1–66.3 °C [58]. ^1^H-NMR (CDCl_3_, 500.1 MHz): δ = 7.25–7.34 (m, 5H, Ar), 5.69 (s, 1H, NH), 4.44 (d, *J* = 5.69 Hz, 2H, CH2), 2.20 (t, *J* = 7.86 Hz, 2H, CH2CO), 1.62–1.68 (m, 2H, CH2), 1.27–1.33 (m, 8H, CH2CH2CH2CH2), 0.87 (t, *J* = 7.16 Hz, 3H, CH3), ^13^C-NMR (CDCl_3_, 125 MHz): δ = 14.05, 22.59, 25.77, 28.99, 29.27, 29.69, 31.68, 36.84, 43.60, 127.50, 127.84, 128.71, 138.45, 172.93.

#### 3.2.2. N-Benzylhexanamide (**13**)

CAS number: 6283-98-3, yellowish white solid, mp = 55.1–55.5 °C [59]. ^1^H-NMR (CDCl_3_, 500.1 MHz): δ = 7.25–7.33 (m, 5H, Ar), 5.84 (s, 1H, NH), 4.42 (d, *J* = 5.73 Hz, 2H, CH2), 2.19 (t, *J* = 7.87 Hz, 2H, CH2CO), 1.62–1.68 (m, 2H, CH2), 1.27–1.34 (m, 4H, CH2CH2), 0.88 (t, *J* = 7.04 Hz, 3H, CH3), ^13^C-NMR (CDCl_3_, 125 MHz): δ = 13.92, 22.39, 25.45, 29.69, 31.48, 36.75, 43.56, 53.42, 127.46, 127,80, 128.68, 138.47, 173.03.

#### 3.2.3. N-Enzylbutyramide (**14**)

CAS number: 10264-14-9, yellowish solid, mp = 43.0–43.6 °C [60]. ^1^H-NMR (CDCl_3_, 500.1 MHz): δ = 7.23–7.31 (m, 5H, Ar), 6.14 (s, 1H, NH), 4.39 (d, *J* = 5.75 Hz, 2H, CH2), 2.16 (t, *J* = 7.64 Hz, 2H, CH2CO), 1.62–1.69 (m, 2H, CH2), 0.93 (t, *J* = 7.42 Hz, 3H, CH3), ^13^C-NMR (CDCl_3_, 125 MHz): δ = 13.78, 19.18, 29.69, 38.58, 43.46, 53.45, 127.38, 127.73, 128.63, 138.54, 173.00.

#### 3.2.4. N-Benzyl-4-phenylbutanamide (**15**)

CAS number: 179923-27-4, yellowish solid, mp = 79.2–80.2 °C [61]. ^1^H-NMR (CDCl_3_, 500.1 MHz): δ = 7.13–7.32 (m, 10H, Ar), 5.84 (s, 1H, NH), 4.39 (d, *J* = 5.73 Hz, 2H, CH2), 2.63 (t, *J* = 7.54 Hz, 2H, CH2), 2.18 (t, *J* = 7.78 Hz, 2H, CH2CO), 1.94–2.00 (m, 2H, CH2), ^13^C-NMR (CDCl_3_, 125 MHz): δ = 27.13, 29.72, 35.22, 35.85, 43.59, 46.42, 70.52, 72.51, 125.99, 127.51, 127.83, 128.41, 128.51, 128.71, 138.41, 141.48, 172, 57.

#### 3.2.5. N-Allyloctanamide (**16**)

CAS number: 70659-85-7, yellow solid, mp = 27.6–28.3 °C [62]. ^1^H-NMR (CDCl_3_, 500.1 MHz): δ = 5.84 (ddt, *J* = 15.97 Hz, 10.29 Hz, 5.09 Hz, 1H, CH), 5.53 (s, 1H, NH), 5.11–5.19 (m, 2H, CH2), 3.88 (t, *J* = 5.73 Hz, 2H, CH2), 2.19 (t, *J* = 7.79 Hz, 2H, CH2CO), 1.61–1.66 (m, 2H, CH2), 1,25–1.31 (m, 8H, CH2CH2CH2CH2), 0.87 (t, *J* = 7.03 Hz, 3H, CH3), ^13^C-NMR (CDCl_3_, 125 MHz): δ = 14.04, 22.59, 25.77, 29.00, 29.27, 31.67, 36.81, 41.86, 116.27, 134.42, 172.94.

#### 3.2.6. N-Allylhexanamide (**17**)

CAS number: 128007-44-3, yellowish oil. ^1^H-NMR (CDCl_3_, 500.1 MHz): δ = 5.84 (ddt, *J* = 15.96 Hz, 10.27 Hz, 5.04 Hz, 1H, CH), 5.55 (s, 1H, NH), 5.11–5.20 (m, 2H, CH2), 3.88 (t, *J* = 5.74 Hz, 2H, CH2), 2.19 (t, *J* = 7.81 Hz, 2H, CH2CO), 1.61–1.67 (m, 2H, CH2), 1.29–1.35 (m, 4H, CH2CH2), 0.89 (t, *J* = 7.08 Hz, 3H, CH3), ^13^C-NMR (CDCl_3_, 125 MHz): δ = 13.91, 22.39, 25.45, 31.47, 36.77, 41.86, 116.29, 134.41, 172.97.

#### 3.2.7. N-Allylbutyramide (**18**)

CAS number: 2978-29-2, yellowish oil. ^1^H-NMR (CDCl_3_, 500.1 MHz): δ = 5.84 (ddt, *J* = 16.00 Hz, 10.32 Hz, 5.06 Hz, 1H, CH), 5.46 (s, 1H, NH), 5.12–5.20 (m, 2H, CH2), 3.89 (t, *J* = 5.74 Hz, 2H, CH2), 2.17 (t, *J* = 7.68 Hz, 2H, CH2CO), 1.64–1.71 (m, 2H, CH2), 0.96 (t, *J* = 7.36 Hz, 3H, CH3), ^13^C-NMR (CDCl_3_, 125 MHz): δ = 13.77, 19.16, 29.69, 38.72, 41.86, 116.33, 134.40.

#### 3.2.8. N-Allyl-4-phenylbutanamide (**19**)

CAS number: 430450-20-7, yellow oil. ^1^H-NMR (CDCl_3_, 500.1 MHz): δ = 7.17–7.29 (m, 5H, Ar), 5.83 (ddt, *J* = 16.00 Hz, 10.28 Hz, 5.06 Hz, 1H, CH), 5.40 (s, 1H, NH), 5.11–5.19 (m, 2H, CH2), 3.87 (t, *J* = 5.77 Hz, 2H, CH2), 2.66 (t, *J* = 7.53 Hz, 2H, CH2), 2.19 (t, *J* = 7.84 Hz, 2H, CH2CO), 1.94–2.02 (m, 2H, CH2), ^13^C-NMR (CDCl_3_, 125 MHz): δ = 27.08, 29.70, 35.19, 35.86, 41.91, 116.44, 125.98, 128.40, 128.49, 134.30, 141.46, 172.42.

#### 3.2.9. N-(Prop-2-yn-1-yl)octanamide (**20**)

CAS number: 422284-34-2, white solid, mp = 72.4–73.4 °C [63]. ^1^H-NMR (CDCl_3_, 500.1 MHz): δ = 5.60 (s, 1H, NH), 4.06 (dd, *J* = 5.18 Hz, *J* = 2.53 Hz 1H, CH2), 4.04 (dd, *J* = 5.18 Hz, *J* = 2.53 Hz, 1H, CH2), 2.22 (t, *J* = 2.55 Hz, 1H, CH), 2.19 (t, *J* = 7.77 Hz, 2H, CH2CO), 1.62–1.66 (m, 2H, CH2), 1.25–1.30 (m, 8H, CH2CH2CH2CH2), 0.87 (t, *J* = 7.05 Hz, 3H, CH3), ^13^C-NMR (CDCl_3_, 125 MHz): δ = 14.04, 22.58, 25.54, 28.97, 29.14, 29.20, 31.65, 36.49, 71.53, 79.67, 172.70.

#### 3.2.10. N-(Prop-2-yn-1-yl)hexanamide (**21**)

CAS number: 62899-12-1, white solid, mp = 47.3–48.0 °C [64]. ^1^H-NMR (CDCl_3_, 500.1 MHz): δ = 5.74 (s, 1H, NH), 4.05 (dd, *J* = 5.21 Hz, *J* = 2.54 Hz 1H, CH2), 4.04 (dd, *J* = 5.21 Hz, *J* = 2.54 Hz, 1H, CH2), 2.22 (t, *J* = 2.53 Hz, 1H, CH), 2.19 (t, *J* = 7.84 Hz, 2H, CH2CO), 1.61–1.67 (m, 2H, CH2), 1.29–1.35 (m, 4H, CH2CH2), 0.89 (t, *J* = 6.98 Hz, 3H, CH3), ^13^C-NMR (CDCl_3_, 125 MHz): δ = 13.89, 22.36, 25.23, 29.13, 31.40, 36.42, 71.49, 79.68, 172.80.

#### 3.2.11. N-(Prop-2-yn-1-yl)butyramide (**22**)

CAS number: 2978-28-1, yellowish solid, mp = 26.1–26.6 °C [65]. ^1^H-NMR (CDCl_3_, 500.1 MHz): δ = 5.56 (s, 1H, NH), 4.06 (dd, *J* = 5.21 Hz, *J* = 2.54 Hz 1H, CH2), 4.05 (dd, *J* = 5.19 Hz, *J* = 2.52 Hz, 1H, CH2), 2.22 (t, *J* = 2.55 Hz, 1H, CH), 2.17 (t, *J* = 7.66 Hz, 2H, CH2CO), 1.64–1.71 (m, 2H, CH2), 0.95 (t, *J* = 7.42 Hz, 3H, CH3), ^13^C-NMR (CDCl_3_, 125 MHz): δ = 13.71, 18.97, 29.14, 29.69, 38.37, 71.55.

#### 3.2.12. 4-Phenyl-N-(prop-2-yn-1-yl)butanamide (**23**)

CAS number: 1250568-47-8, colorless oil. ^1^H-NMR (CDCl_3_, 500.1 MHz): δ = 7.16–7.29 (m, 5H, Ar), 5.54 (s, 1H, NH), 4.04 (dd, *J* = 5.24 Hz, *J* = 2.56 Hz 1H, CH2), 4.03 (dd, *J* = 5.19 Hz, *J* = 2.51 Hz, 1H, CH2), 2.66 (t, *J* = 7.53 Hz, 2H, CH2), 2.22 (t, *J* = 2.58 Hz, 1H, CH), 2.19 (t, *J* = 7.83 Hz, 2H, CH2CO), 1.95–2.01 (m, 2H, CH2), ^13^C-NMR (CDCl_3_, 125 MHz): δ = 26.84, 29.15, 29.17, 35.08, 35.49, 71.59, 79.58, 126.02, 128.42, 128.50, 141.33, 172.22.

#### 3.2.13. 1-(Piperidin-1-yl)octan-1-one (**24**)

CAS number: 20299-83-6, colorless oil. ^1^H-NMR (CDCl_3_, 500.1 MHz): δ = 3.54 (t, *J* = 5.47 Hz, 2H, CH2N), 3.39 (t, *J* = 5.36 Hz, 2H, CH2N), 2.30 (t, *J* = 7.91 Hz, 2H, CH2CO), 1.50–1.66 (m, 8H, CH2CH2CH2CH2), 1.25–1.32 (m, 8H, CH2CH2CH2CH2), 0.87 (t, *J* = 7.01 Hz, 3H, CH3), ^13^C-NMR (CDCl_3_, 125 MHz): δ = 14.05, 22.60, 24.60, 25.50, 25.59, 26.58, 29.10, 29.50, 31.72, 33.49, 42.58, 46.72, 171.52.

#### 3.2.14. 1-(Piperidin-1-yl)hexan-1-one (**25**)

CAS number: 15770-38-4, yellowish oil. ^1^H-NMR (CDCl_3_, 500.1 MHz): δ = 3.54 (t, *J* = 5.47 Hz, 2H, CH2N), 3.39 (t, *J* = 5.47 Hz, 2H, CH2N), 2.30 (t, *J* = 7.77 Hz, 2H, CH2CO), 1.51–1.65 (m, 8H, CH2CH2CH2CH2), 1.30–1.35 (m, 4H, CH2CH2), 0.90 (t, *J* = 6.91 Hz, 3H, CH3), ^13^C-NMR (CDCl_3_, 125 MHz): δ = 13.96, 22.49, 24.61, 25.18, 25.60, 26.59, 31.72, 33.45, 42.59, 46.72, 171.53.

#### 3.2.15. 1-(Piperidin-1-yl)butan-1-one (**26**)

CAS number: 4637-70-1, yellow oil. ^1^H-NMR (CDCl_3_, 500.1 MHz): δ = 3.54 (t, *J* = 5.48 Hz, 2H, CH2N), 3.39 (t, *J* = 5.34 Hz, 2H, CH2N), 2.29 (t, *J* = 7.78 Hz, 2H, CH2CO), 1.50–1.69 (m, 8H, CH2CH2CH2CH2), 0.96 (t, *J* = 7.41 Hz, 3H, CH3), ^13^C-NMR (CDCl_3_, 125 MHz): δ = 14.04, 18.88, 24.61, 25.61, 26.58, 35.40, 42.58, 46.71, 171.34.

#### 3.2.16. 4-Phenyl-1-(piperidin-1-yl)butan-1-one (**27**)

CAS number: 41208-51-9, white solid, mp = 153.9–154.4 °C [66]. ^1^H-NMR (CDCl_3_, 500.1 MHz): δ = 7.16–7.29 (m, 5H, Ar), 3.54 (t, *J* = 5.53 Hz, 2H, CH2N), 3.31 (t, *J* = 5.47 Hz, 2H, CH2N), 2.67 (t, *J* = 7.66 Hz, 2H, CH2), 2.32 (t, *J* = 7.84 Hz, 2H, CH2CO), 1.93–1.99 (m, 2H, CH2), 1.49–1.64 (m, 6H, CH2CH2CH2), ^13^C-NMR (CDCl_3_, 125 MHz): δ = 24.58, 25.60, 26.54, 26.84, 29.69, 33.54, 35.42, 42.63, 46.61, 125.86, 128.33, 128.49, 141.85, 171.01.

#### 3.2.17. 1-Morpholinooctan-1-one (**28**)

CAS number: 5338-65-8, colorless oil. ^1^H-NMR (CDCl_3_, 500.1 MHz): δ = 3.66 (t, *J* = 5.14 Hz, 4H, CH2OCH2), 3.61 (t, *J* = 3.75 Hz, 2H, CH2N), 3.46 (t, *J* = 4.53 Hz, 2H, CH2N), 2.30 (t, *J* = 7.83 Hz, 2H, CH2CO), 1.59–1.65 (m, 2H, CH2), 1.25–1.34 (m, 8H, CH2CH2CH2CH2), 0.88 (t, *J* = 7.08 Hz, 3H, CH3), ^13^C-NMR (CDCl_3_, 125 MHz): δ = 14.03, 22.58, 25.25, 29.05, 29.40, 31.68, 33.11, 41.85, 46.06, 66.68, 66.95, 171.89.

#### 3.2.18. 1-Morpholinohexan-1-one (**29**)

CAS number: 17598-10-6, yellowish oil. ^1^H-NMR (CDCl_3_, 500.1 MHz): δ = 3.66 (t, *J* = 5.18 Hz, 4H, CH2OCH2), 3.61 (t, *J* = 3.75 Hz, 2H, CH2N), 3.46 (t, *J* = 4.56 Hz, 2H, CH2N), 2.30 (t, *J* = 7.85 Hz, 2H, CH2CO), 1.60–1.66 (m, 2H, CH2), 1.30–1.36 (m, 4H, CH2CH2), 0.90 (t, *J* = 6.95 Hz, 3H, CH3), ^13^C-NMR (CDCl_3_, 125 MHz): δ = 13.92, 22.45, 24.93, 31.62, 33.07, 41.86, 46.06, 66.68, 66.96, 171.90.

#### 3.2.19. 1-Morpholinobutan-1-one (**30**)

CAS number: 5327-51-5, colorless oil. ^1^H-NMR (CDCl_3_, 500.1 MHz): δ = 3.66 (t, *J* = 5.20 Hz, 4H, CH2OCH2), 3.61 (t, *J* = 4.56 Hz, 2H, CH2N), 3.46 (t, *J* = 4.56 Hz, 2H, CH2N), 2.29 (t, *J* = 7.70 Hz, 2H, CH2CO), 1.63–1.70 (m, 2H, CH2), 0.97 (t, *J* = 7.46 Hz, 3H, CH3), ^13^C-NMR (CDCl_3_, 125 MHz): δ = 13.96, 18.66, 35.02, 41.86, 46.05, 66.69, 66.97, 171.75.

#### 3.2.20. 1-Morpholino-4-phenylbutan-1-one (**31**)

CAS number: 61123-44-2, white solid, mp = 40.7–42.5 °C [67]. ^1^H-NMR (CDCl_3_, 500.1 MHz): δ = 7.17–7.29 (m, 5H, Ar), 3.59–3.66 (m, 6H, NCH2CH2OCH2), 3.37 (t, *J* = 4.68 Hz, 2H, NCH2), 2.68 (t, *J* = 7.59 Hz, 2H, CH2), 2.30 (t, *J* = 7.71 Hz, 2H, CH2CO), 1.95–2.01 (m, 2H, CH2), ^13^C-NMR (CDCl_3_, 125 MHz): δ = 26.54, 29.70, 32.10, 35.26, 45.89, 45.92, 66.63, 66.95, 125.98, 128.40, 128.48, 141.57, 171.42.

#### 3.2.21. N-(2-(Dimethylamino)ethyl)octanamide (**32**)

CAS number: 114011-26-6, colorless oil. ^1^H-NMR (CDCl_3_, 500.1 MHz): δ = 6.14 (s, 1H, NH), 3.30–3.34 (m, 2H, CONHCH2), 2.41 (t, *J* = 5.90 Hz, 2H, CH2), 2.23 (s, 6H, CH3CH3), 2.17 (t, *J* = 7.82 Hz, 2H, CH2CO), 1.59–1.65 (m, 2H, CH2), 1.27–1.30 (m, 8H, CH2CH2CH2CH2), 0.87 (t, *J* = 7.09 Hz, 3H, CH3), ^13^C-NMR (CDCl_3_, 125 MHz): δ = 14.04, 22.58, 25.78, 29.00, 29.26, 29.68, 31.69, 36.64, 36.73, 45.07, 57.94, 173.37.

#### 3.2.22. N-(2-(Dimethylamino)ethyl)hexanamide (**33**)

CAS number: 114011-25-5, colorless oil. ^1^H-NMR (CDCl_3_, 500.1 MHz): δ = 6.07 (s, 1H, NH), 3.31–3.34 (m, 2H, CONHCH2), 2.41 (t, *J* = 5.90 Hz, 2H, CH2), 2.23 (s, 6H, CH3CH3), 2.17 (t, *J* = 7.81 Hz, 2H, CH2CO), 1.60–1.66 (m, 2H, CH2), 1.28–1.36 (m, 4H, CH2CH2), 0.89 (t, *J* = 7.04 Hz, 3H, CH3), ^13^C-NMR (CDCl_3_, 125 MHz): δ = 13.94, 22.41, 25.46, 29.69, 31.49, 36.62, 36.72, 45.08, 57.91, 173.30.

#### 3.2.23. N-(2-(Dimethylamino)ethyl)butyramide (**34**)

CAS number: 63224-16-8, yellowish oil. ^1^H-NMR (CDCl_3_, 500.1 MHz): δ = 6.12 (s, 1H, NH), 3.31–3.35 (m, 2H, CONHCH2), 2.42 (t, *J* = 5.88 Hz, 2H, CH2), 2.24 (s, 6H, CH3CH3), 2.15 (t, *J* = 7.69 Hz, 2H, CH2CO), 1.62–1.70 (m, 2H, CH2), 0.94 (t, *J* = 7.36 Hz, 3H, CH3), ^13^C-NMR (CDCl_3_, 125 MHz): δ = 13.77, 19.19, 29.69, 36.57, 38.65, 45.03, 57.19, 173.14.

#### 3.2.24. N-(2-(Dimethylamino)ethyl)-4-phenylbutanamide (**35**)

CAS number: 63224-25-9, yellowish oil. ^1^H-NMR (CDCl_3_, 500.1 MHz): δ = 7.17–7.28 (m, 5H, Ar), 3.30–3.33 (m, 2H, CONHCH2), 2.65 (t, *J* = 7.60 Hz, 2H, CH2), 2.40 (t, *J* = 5.88 Hz, 2H, CH2), 2.22 (s, 6H, CH3CH3), 2.18 (t, *J* = 7.79 Hz, 2H, CH2CO), 1.94–2.00 (m, 2H, CH2), ^13^C-NMR (CDCl_3_, 125 MHz): δ = 22.68, 27.13, 29.35, 29.69, 35.23, 25.84, 36.66, 45.07, 57.90, 125.91, 128.35, 128.51, 141.61, 172.85.

#### 3.2.25. N-(3-(Dimethylamino)propyl)ctanamide (**36**)

CAS number: 22890-10-4, colorless oil. ^1^H-NMR (CDCl_3_, 500.1 MHz): δ = 6.89 (s, 1H, NH), 3.30–3.34 (m, 2H, CONHCH2), 2.37 (t, *J* = 6.41 Hz, 2H, CH2), 2.23 (s, 6H, CH3CH3), 2.14 (t, *J* = 7.80 Hz, 2H, CH2CO), 1.57–1.68 (m, 4H, CH2CH2), 1.25–1.30 (m, 8H, CH2CH2CH2CH2), 0.87 (t, *J* = 7.04 Hz, 3H, CH3), ^13^C-NMR (CDCl_3_, 125 MHz): δ = 14.04, 22.59, 25.75, 26.14, 29.02, 29.26, 29.68, 31.68, 36.96, 39.15, 45.34, 58.50, 173.16.

#### 3.2.26. N-(3-(Dimethylamino)propyl)hexanamide (**37**)

CAS number: 73603-23-3, yellowish oil. ^1^H-NMR (CDCl_3_, 500.1 MHz): δ = 6.92 (s, 1H, NH), 3.30–3.34 (m, 2H, CONHCH2), 2.37 (t, *J* = 6.45 Hz, 2H, CH2), 2.22 (s, 6H, CH3CH3), 2.14 (t, *J* = 7.77 Hz, 2H, CH2CO), 1.57–1.67 (m, 4H, CH2CH2), 1.28–1.34 (m, 4H, CH2CH2), 0.89 (t, *J* = 7.16 Hz, 3H, CH3), ^13^C-NMR (CDCl_3_, 125 MHz): δ = 13.90, 22.40, 25.42, 26.17, 29.67, 31.45, 36.89, 39.15, 45.35, 58.49, 173.18.

#### 3.2.27. N-(3-(Dimethylamino)propyl)butyramide (**38**)

CAS number: 53201-67-5, colorless oil. ^1^H-NMR (CDCl_3_, 500.1 MHz): δ = 6.89 (s, 1H, NH), 3.32–3.35 (m, 2H, CONHCH2), 2.43 (t, *J* = 6.43 Hz, 2H, CH2), 2.27 (s, 6H, CH3CH3), 2.13 (t, *J* = 7.63 Hz, 2H, CH2CO), 1.60–1.70 (m, 4H, CH2CH2), 0.94 (t, *J* = 7.39 Hz, 3H, CH3), ^13^C-NMR (CDCl_3_, 125 MHz): δ = 13.80, 19.12, 26.01, 29.69, 38.87, 38.98, 45.16, 58.36, 172.97.

#### 3.2.28. N-(3-(Dimethylamino)propyl)-4-phenylbutanamide (**39**)

CAS number: 885912-19-6, yellowish oil. ^1^H-NMR (CDCl_3_, 500.1 MHz): δ = 7.16–7.28 (m, 5H, Ar), 6.88 (s, 1H, NH), 3.30–3.34 (m, 2H, CONHCH2), 2.64 (t, *J* = 7.64 Hz, 2H, CH2), 2.38 (t, *J* = 6.42 Hz, 2H, CH2), 2.22 (s, 6H, CH3CH3), 2.16 (t, *J* = 7.78 Hz, 2H, CH2CO), 1.92–1.98 (m, 2H, CH2), 1.62–1.67 (m, 2H, CH2), ^13^C-NMR (CDCl_3_, 125 MHz): δ = 14.11, 26.11, 27.19, 29.69, 35.25, 36.09, 39.17, 45.30, 53.42, 58.52, 125.89, 128.34, 128.46, 141.64, 172.61.

## 4. Conclusions

We successfully developed a sustainable and green enzymatic strategy for the synthesis of amides from free carboxylic acids and amines by using *Candida antarctica* lipase B as a biocatalyst, using GC-MS analysis to monitor the reaction progress. The green enzymatic amidation is simple and efficient without any additives, with the application of cyclopentyl methyl ether as the solvent, which is a greener and safer solvent alternative in comparison with the usual organic solvents. The scope of the reaction was extended to the preparation of 28 diverse amides, by using four different free carboxylic acids and seven amines, including primary and secondary amines as well as cyclic amines. In every case, excellent conversions and yields were achieved without the need of any intensive purification step. This enzymatic methodology offers a way to synthetize pure amides. That is, this synthetic approach is both eco-friendly and practical for large-scale production.

## Figures and Tables

**Table 1:** Substrate scope of amide formation in CPME solvent with conversion data.

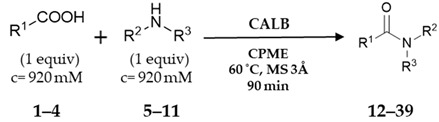				
Substrates	Octanoic Acid (1)	Hexanoic Acid (2)	Butyric Acid (3)	4-Phenylbutyric Acid (4)
benzylamine (**5**)	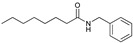 **12**, 99%	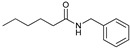 **13**, 98%,	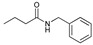 **14**, 98%	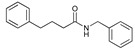 **15**, 99%
allylamine (**6**)	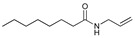 **16**, 96%	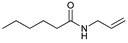 **17**, 96%	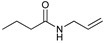 **18**, 94%	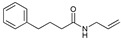 **19**, 98%
propargylamine (**7**)	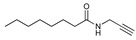 **20**, 97%	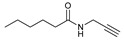 **21**, 98%	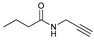 **22**, 92%	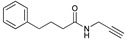 **23**, 97%
piperidine (**8**)	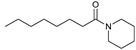 **24**, 95%	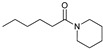 **25**, 93%	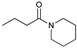 **26**, 92%	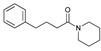 **27**, 97%
morpholine (**9**)	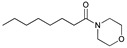 **28**, 99%	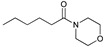 **29**, 98%	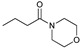 **30**, 94%	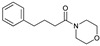 **31**, 96%
*N*^1^,*N*^1^-dimethylethane-1,2-diamine (**10**)	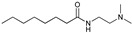 **32**, 96%	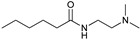 **33**, 98%	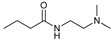 **34**, 93%	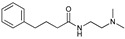 **35**, 97%
*N*^1^,*N*^1^-dimethylpropane-1,3-diamine (**11**)	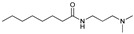 **36**, 99%	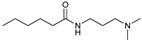 **37**, 98%	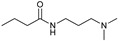 **38**, 92%	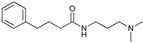 **39**, 99%

## Data Availability

The data presented in this study are available in the article and Supplementary Materials.

## Supplementary Materials

The following supporting information can be downloaded at https://www.mdpi.com/article/10.3390/molecules28155706/s1, Figures S1–S62: ^1^H-NMR and ^13^C-NMR spectra of **12–39** amide products and GC-MS measurement.

## Sample Availability

Samples of compounds **12**–**39** are available from the authors.
