# Supplementary material for: A Sustainable Green Enzymatic Method for Amide Bond Formation
Source: Molecules. 2023 Jul 28;28(15):5706. doi: 10.3390/molecules28155706 (PMC10419938; doi:10.3390/molecules28155706)
Supplement: Supplementary file 1 [file molecules-28-05706-s001.zip › molecules-2425772-supplementary.pdf]

Supplementary Information

A sustainable green enzymatic method for amide bond formation

György Orsy [1], Sayeh Shahmohammadi[1,2], Enikő Forró [1*]

[1] Institute of Pharmaceutical Chemistry, University of Szeged, Eötvös u. 6, H-6720 Szeged, Hungary; orsy.gyorgy@szte.hu (G.O.); sayeh.s@pharm.u-szeged.hu (S.S.)
[2] Stereochemistry Research Group, Eötvös Loránd Research Network, University of Szeged, Eötvös u. 6, Szeged, H-6720, Hungary
* Correspondence: forro.eniko@szte.hu (E.F.); Tel.: +36-62-544964

Contents

1. ^{1}H- and ^{13}C NMR spectra 2
2. GC-MS measurement 30

1. ^{1}H- and ^{13}C NMR spectra

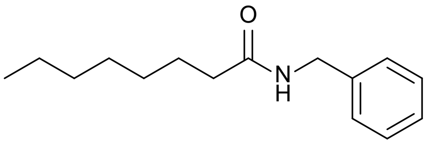

N-benzyloctanamide (**12**)

The compound is obtained as a white solid in 96% yield, mp= 65.1–66.3 ^{0}C (206.0 mg, 0.883 mmol).

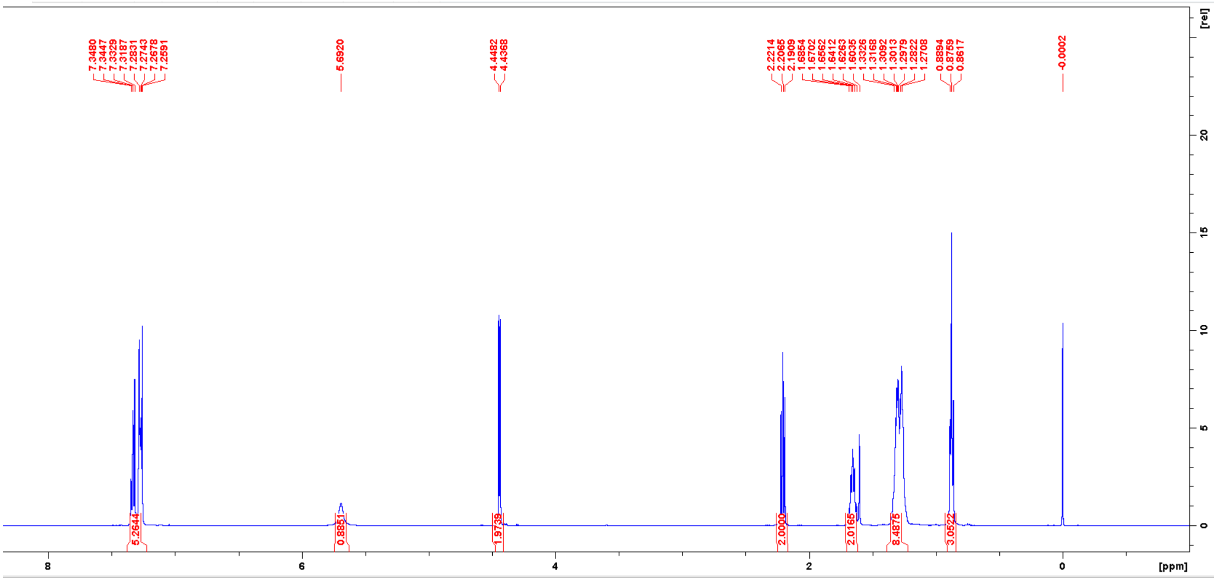

Figure S1. ^{1}H NMR spectrum of *N*-benzyloctanamide (**12**) measured in Chloroform-d, 99.8 atom % D at 296 K.

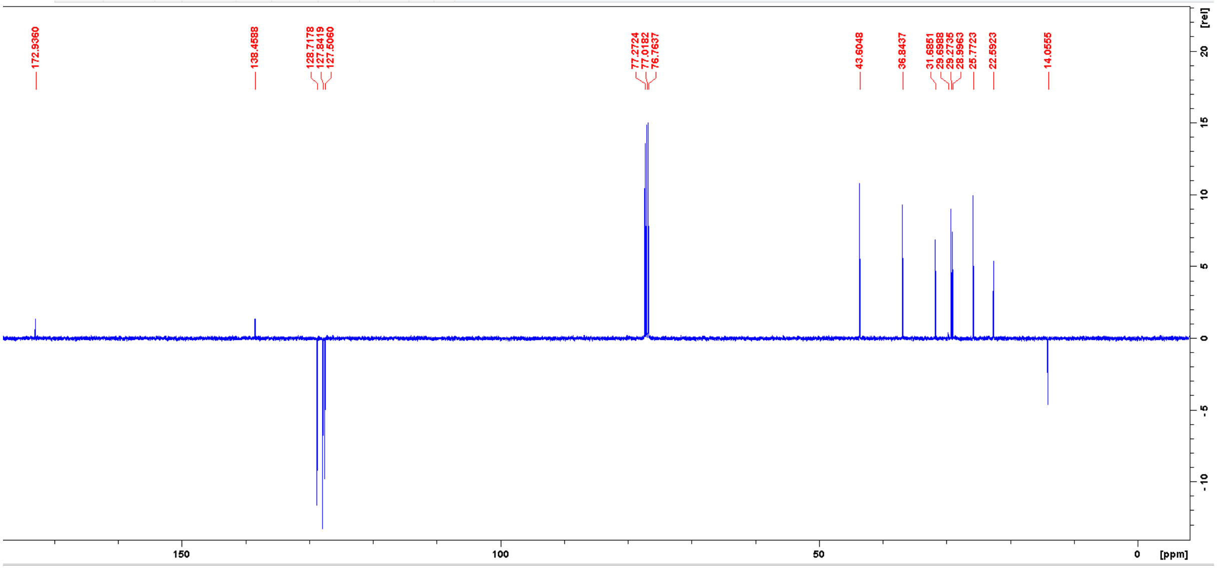

Figure S2. APT NMR spectrum of *N*-benzyloctanamide (**12**) measured Chloroform-d, 99.8 atom % D at 296 K.

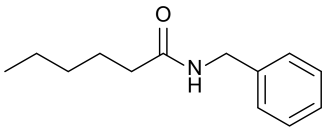

N-benzylhexanamide (**13**)

The compound is obtained as a yellowish white solid in 95% yield, mp= 55.1–55.5 ^{0}C (179.3 mg, 0.874 mmol).

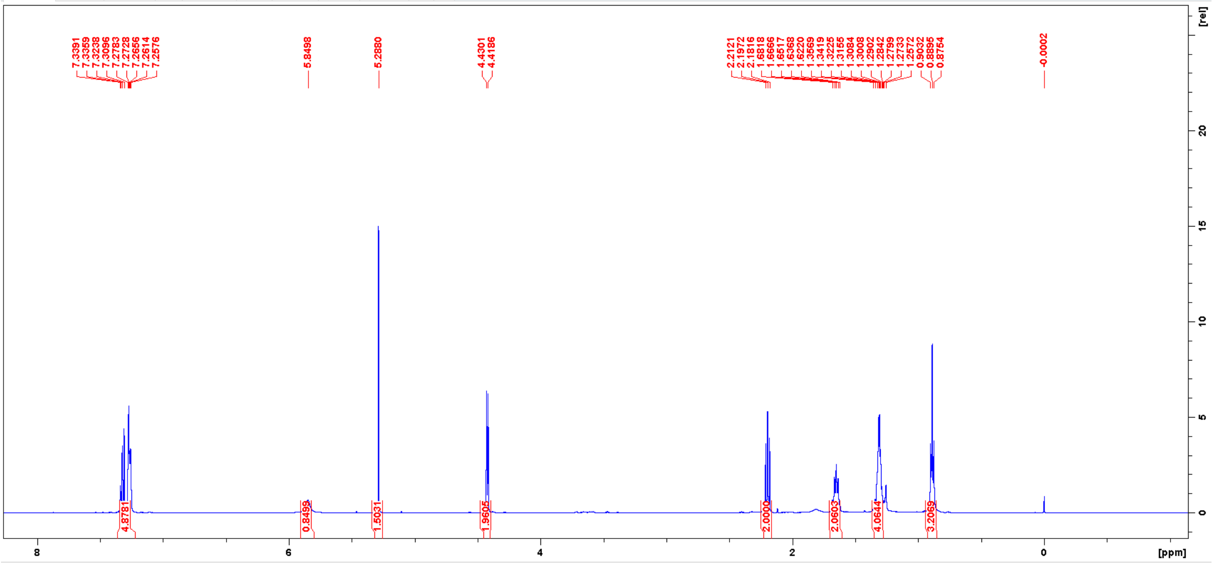

Figure S3. ^{1}H NMR spectrum of *N*-benzylhexanamide (**13**) measured in Chloroform-d, 99.8 atom % D at 296 K.

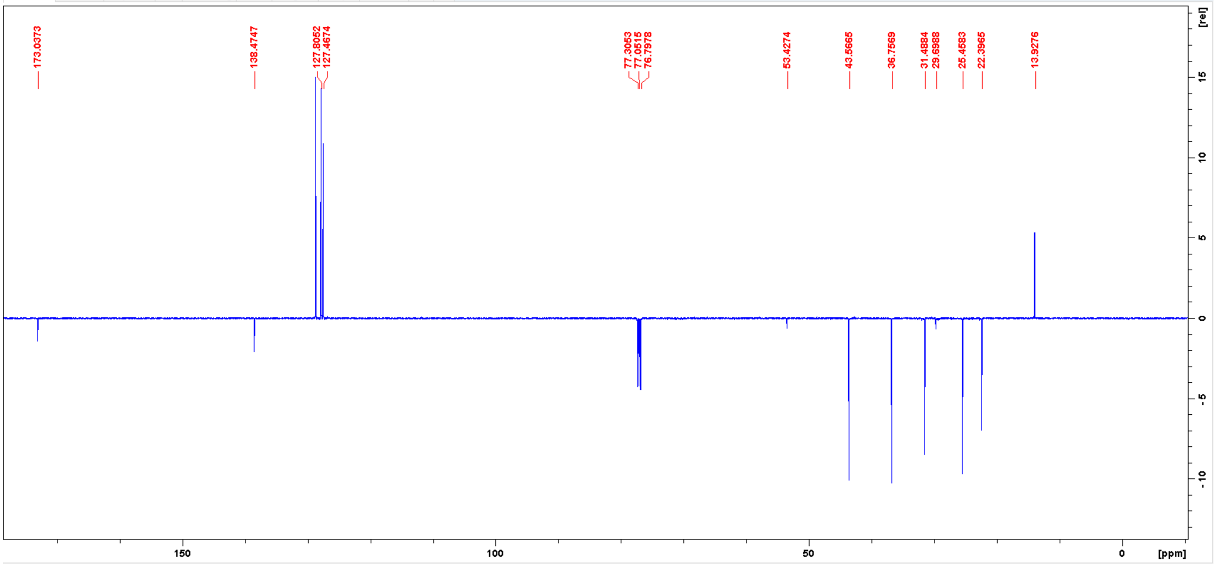

Figure S4. APT NMR spectrum of *N*-benzylhexanamide (**13**) measured Chloroform-d, 99.8 atom % D at 296 K.

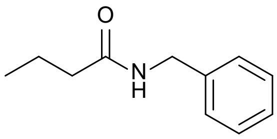

N-benzylbutyramide (**14**)

The compound is obtained as a yellowish solid in 96% yield, mp= 43.0–43.6 ⁰C (156.5 mg, 0.883 mmol).

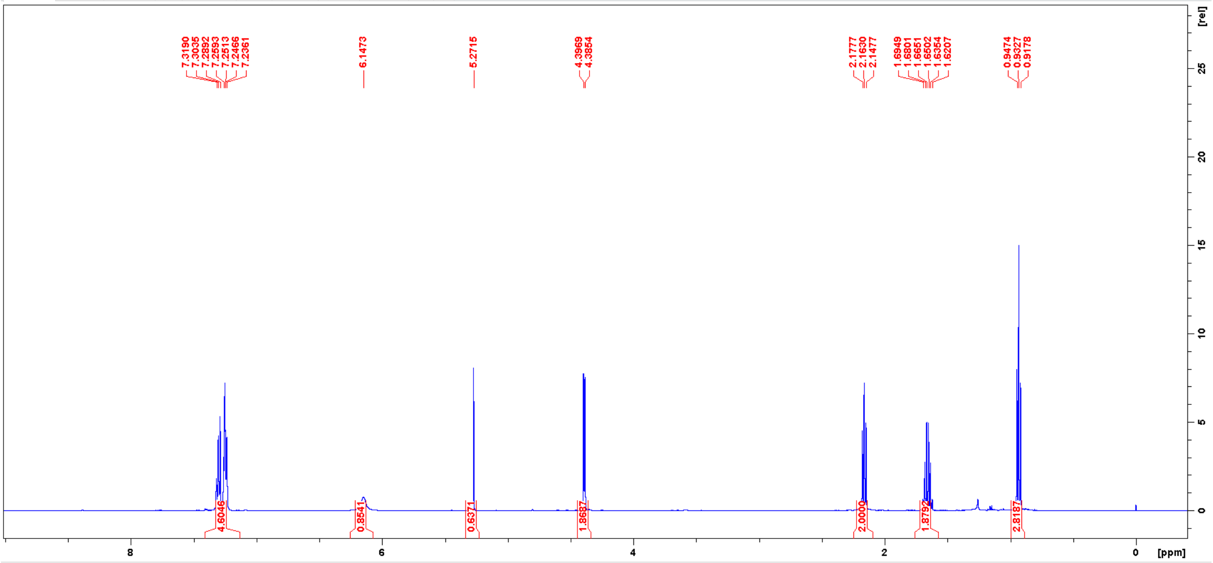

Figure S5. ^{1}H NMR spectrum of *N*-benzylbutyramide (**14**) measured in Chloroform-d, 99.8 atom % D at 296 K.

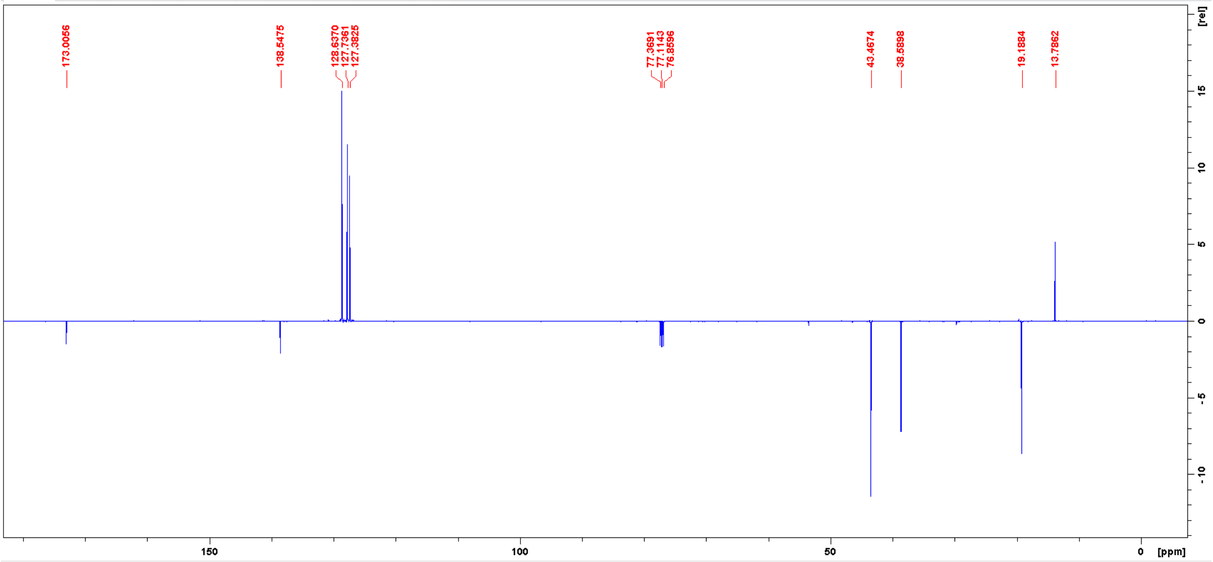

Figure S6. APT NMR spectrum of *N*-benzylbutyramide (**14**) measured Chloroform-d, 99.8 atom % D at 296 K.

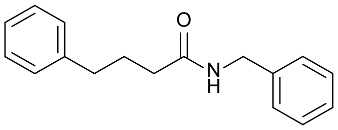

N-benzyl-4-phenylbutanamide (**15**)

The compound is obtained as a yellowish solid in 94% yield, mp= 79.2–80.2 ⁰C (219.1 mg, 0.864 mmol).

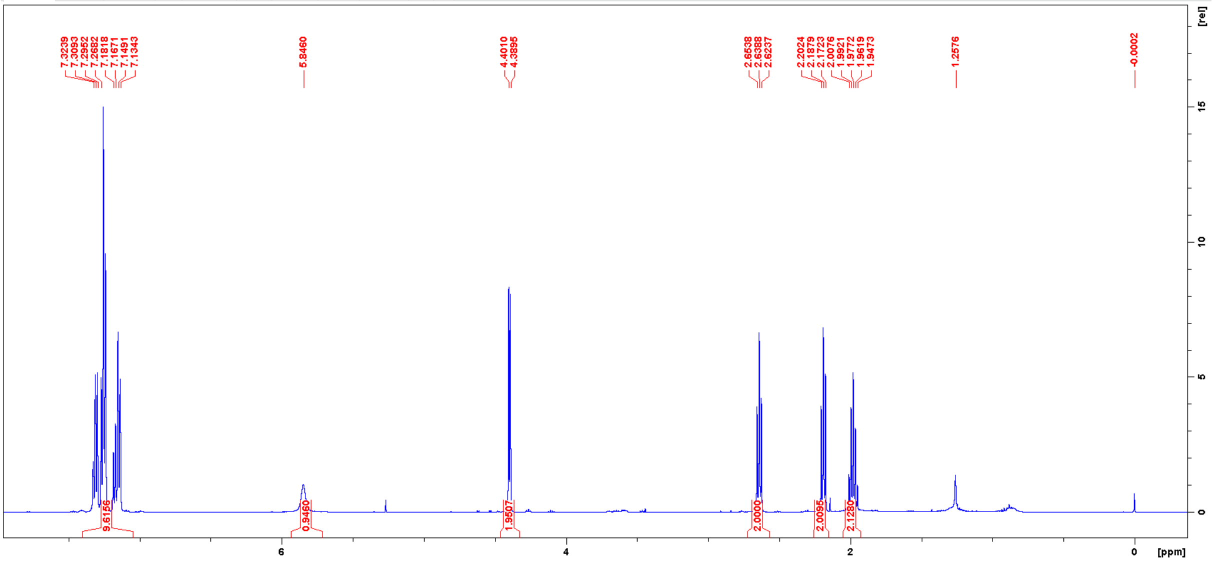

Figure S7. ^{1}H NMR spectrum of *N*-benzyl-4-phenylbutanamide (**15**) measured in Chloroform-d, 99.8 atom % D at 296 K.

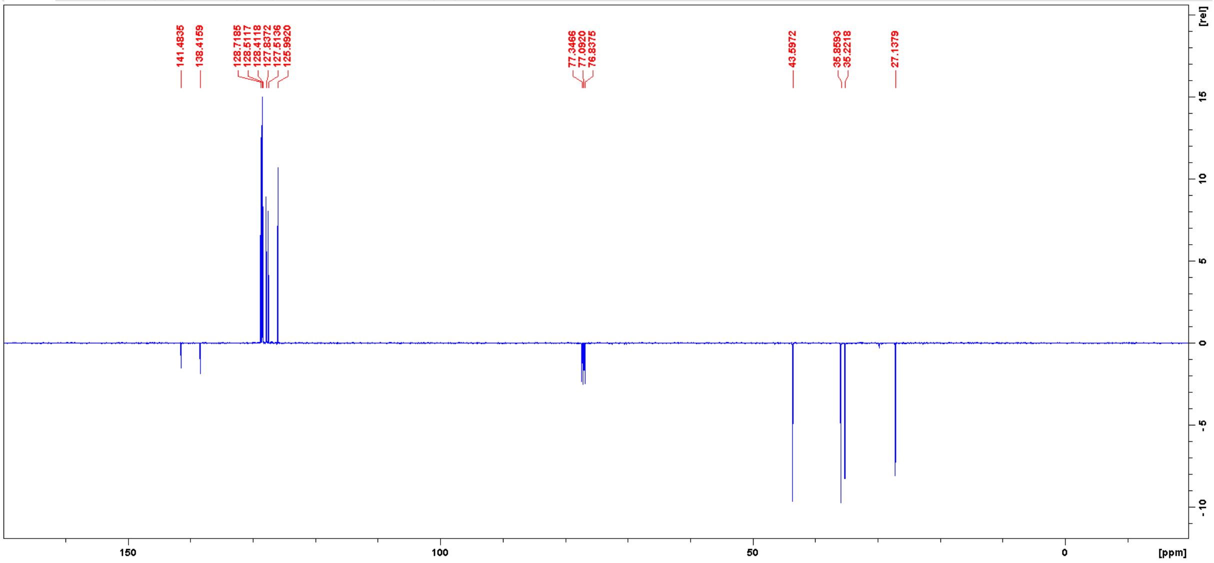

Figure S8. APT NMR spectrum of *N*-benzyl-4-phenylbutanamide (**15**) measured Chloroform-d, 99.8 atom % D at 296 K.

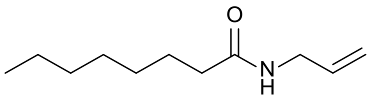

N-allyloctanamide (**16**)

The compound is obtained as a yellow solid in 95% yield, mp= 27.6–28.3 ⁰C (160.1 mg, 0.874 mmol).

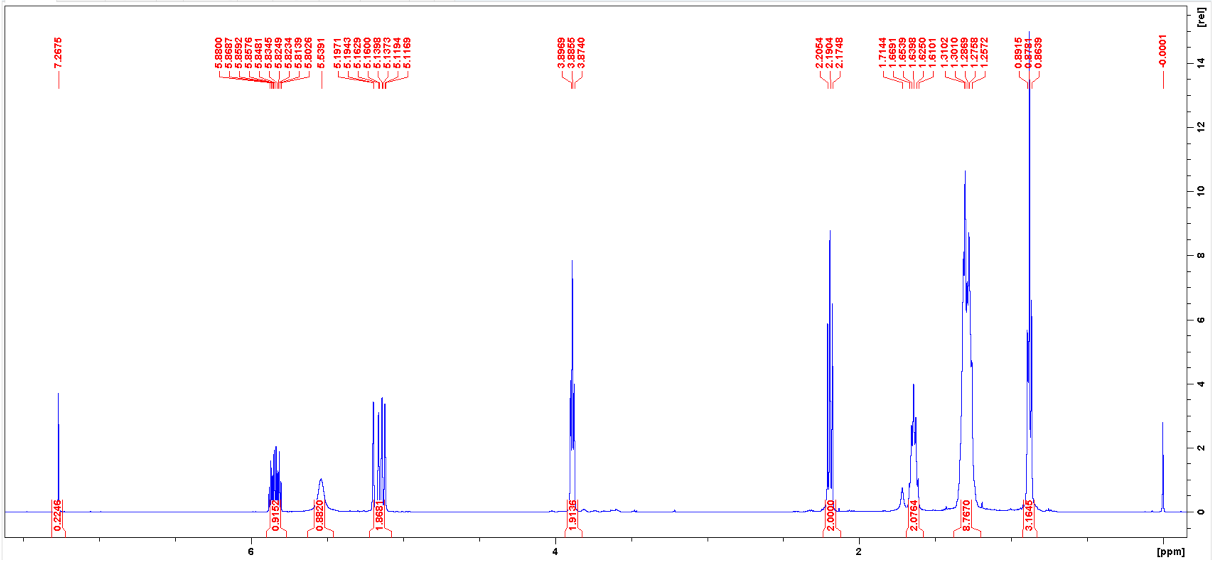

Figure S9. ^{1}H NMR spectrum of *N*-allyloctanamide (**16**) measured in Chloroform-d, 99.8 atom % D at 296 K.

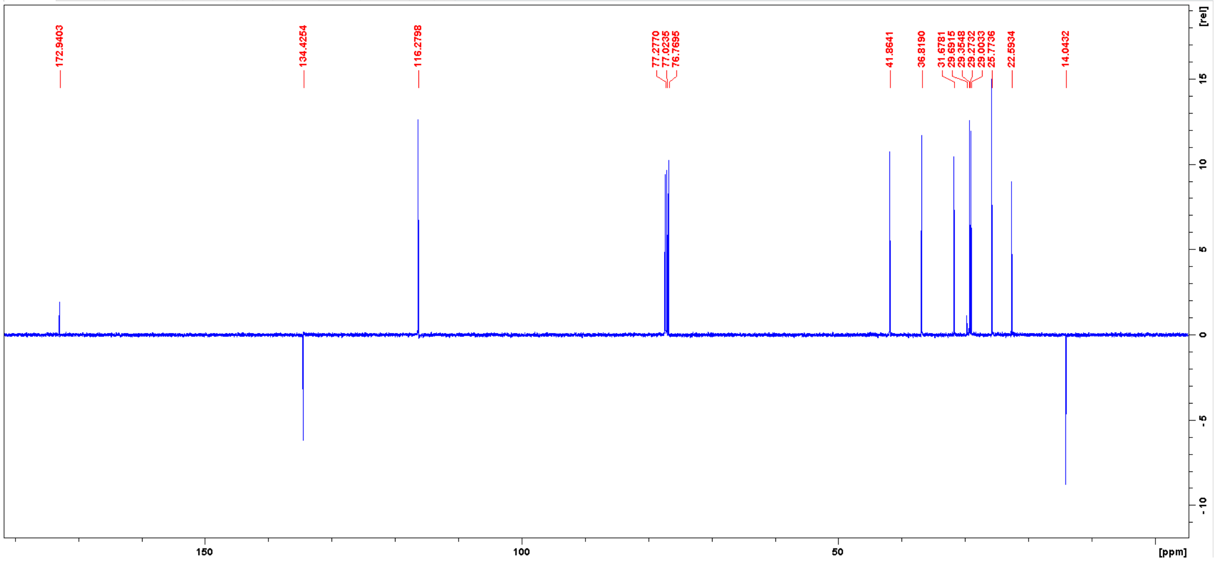

Figure S10. APT NMR spectrum of *N*-allyloctanamide (**16**) measured Chloroform-d, 99.8 atom % D at 296 K.

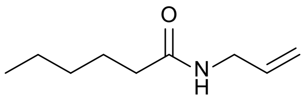

N-allylhexanamide (**17**)

The compound is obtained as a yellowish oil in 96% yield (161.8 mg, 0.883 mmol).

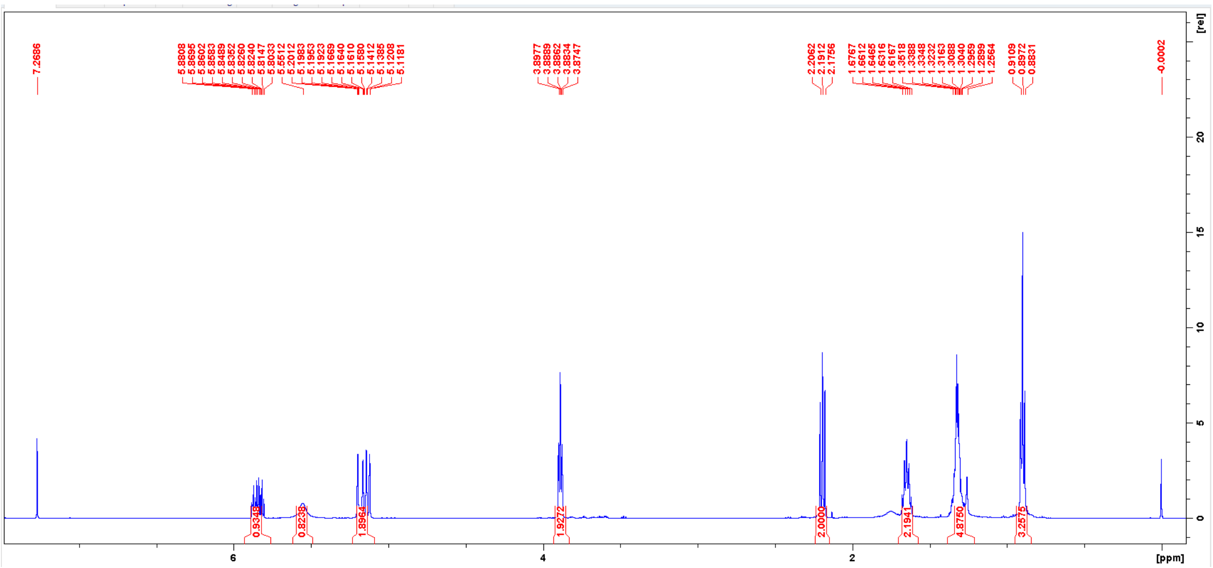

Figure S11. ^{1}H NMR spectrum of *N*-allylhexanamide (**17**) measured in Chloroform-d, 99.8 atom % D at 296 K.

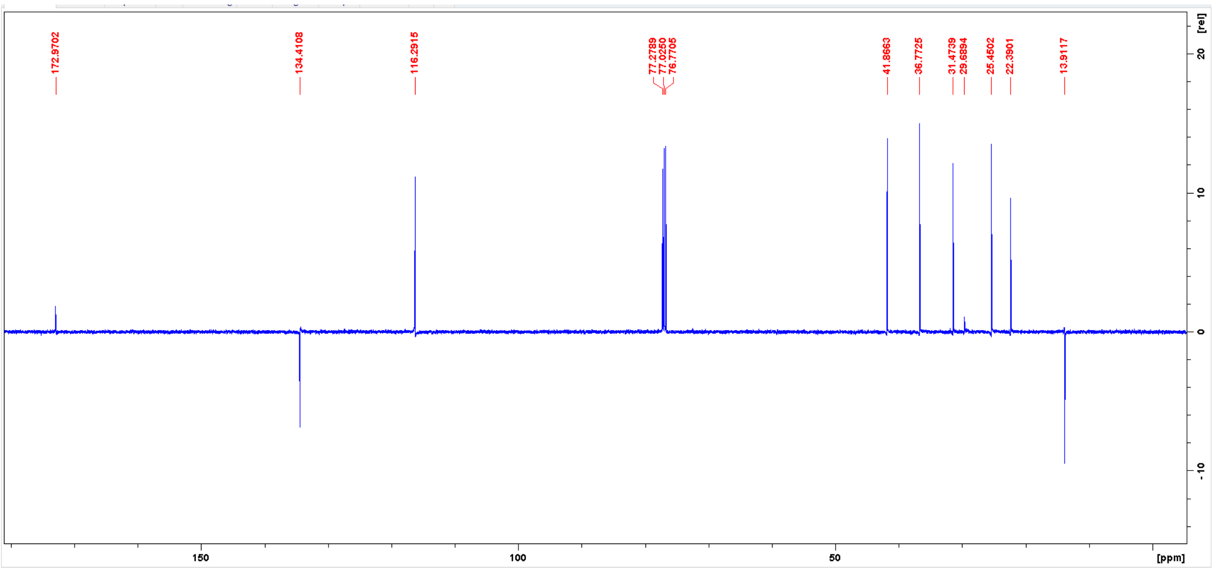

Figure S12. APT NMR spectrum of *N*-allylhexanamide (**17**) measured Chloroform-d, 99.8 atom % D at 296 K.

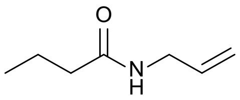

N-allylbutyramide (**18**)

The compound is obtained as a yellowish oil in 92% yield (107.5 mg, 0.846 mmol).

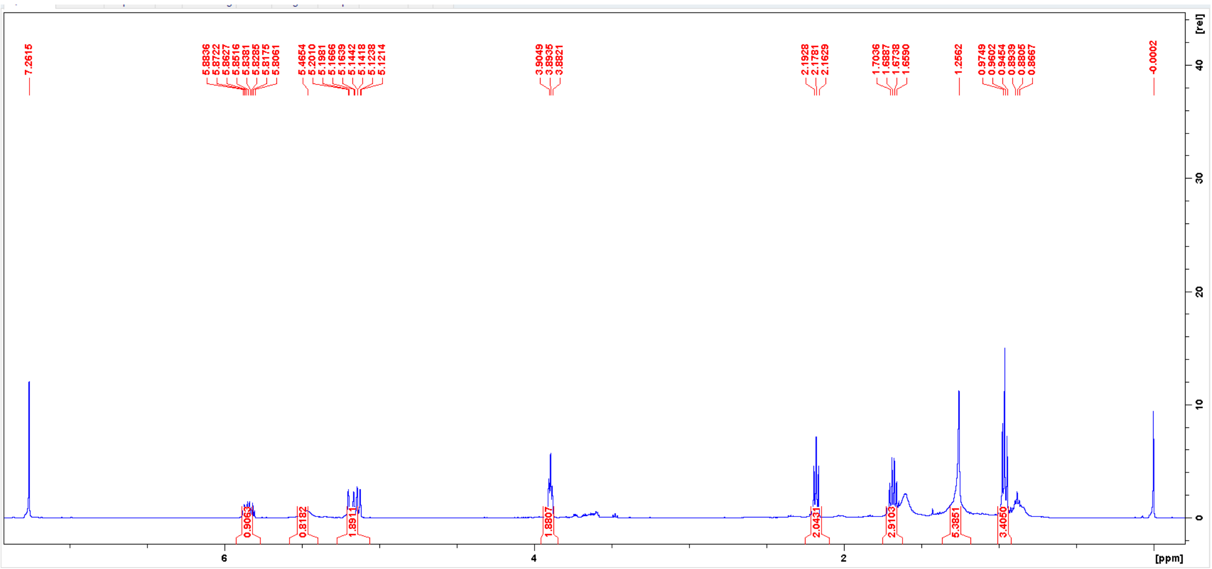

Figure S13. ^{1}H NMR spectrum of *N*-allylbutyramide (**18**) measured in Chloroform-d, 99.8 atom % D at 296 K.

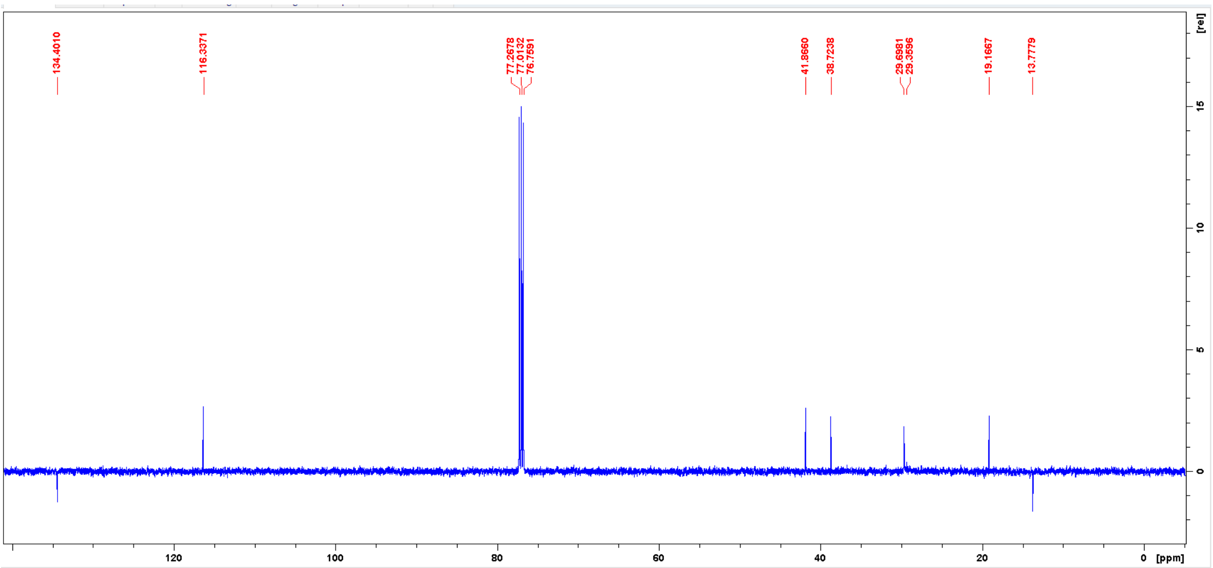

Figure S14. APT NMR spectrum of *N*-allylbutyramide (**18**) measured Chloroform-d, 99.8 atom % D at 296 K.

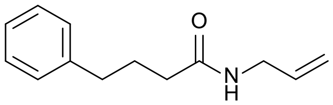

N-allyl-4-phenylbutanamide (**19**)

The compound is obtained as a yellow oil in 96% yield (179.5 mg, 0.883 mmol).

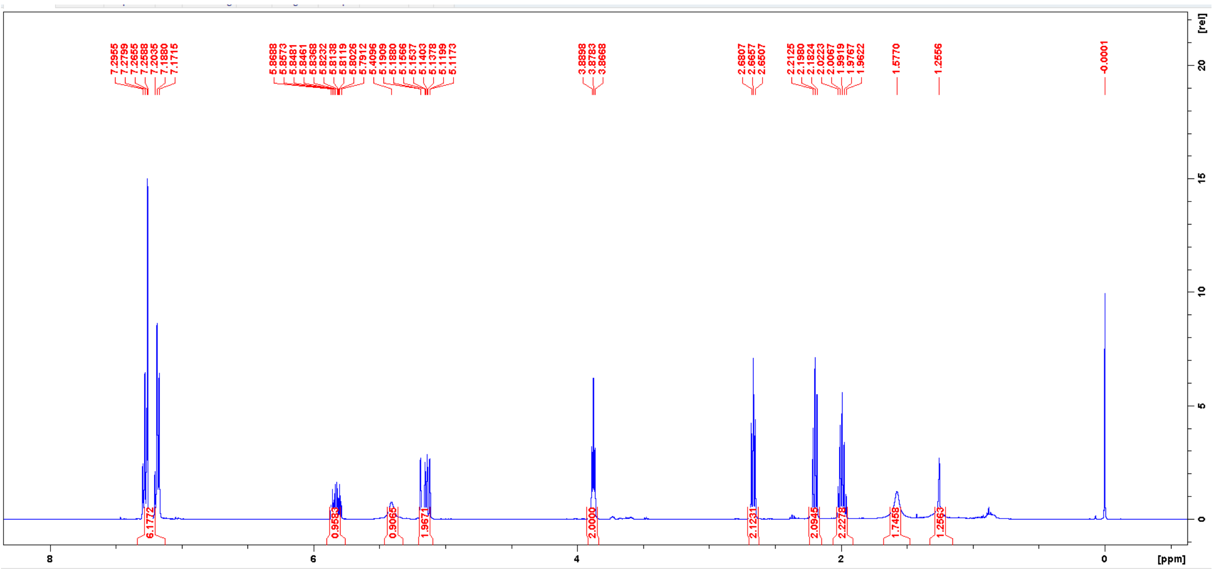

Figure S15. ^{1}H NMR spectrum of *N*-allyl-4-phenylbutanamide (**19**) measured in Chloroform-d, 99.8 atom % D at 296 K.

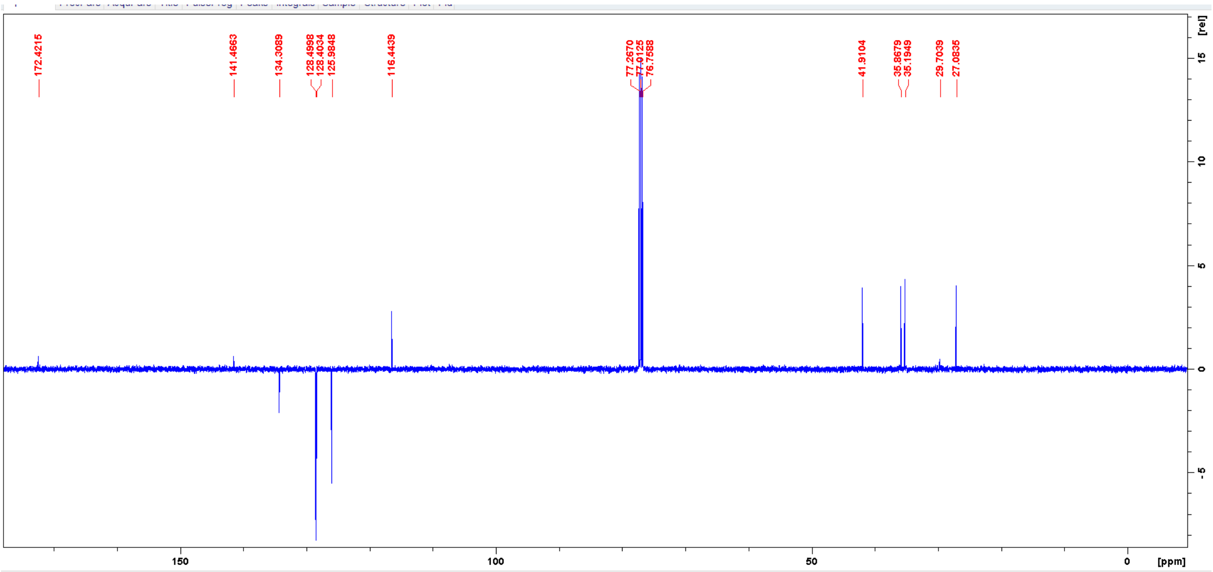

Figure S16. APT NMR spectrum of *N*-allyl-4-phenylbutanamide (**19**) measured Chloroform-d, 99.8 atom % D at 296 K.

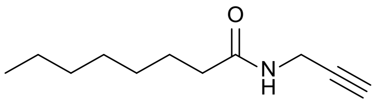

N-(prop-2-yn-1-yl)octanamide (**20**)

The compound is obtained as a white solid in 94% yield, mp= 72.4–73.4 °C (156.7 mg, 0.864 mmol).

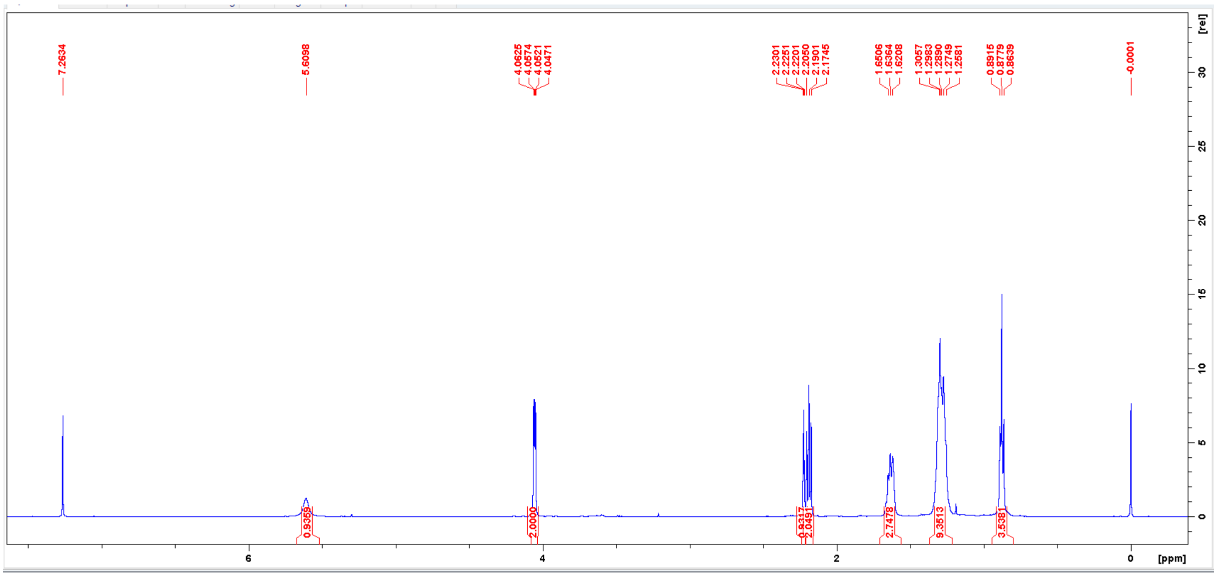

Figure S17. ^{1}H NMR spectrum of *N*-(prop-2-yn-1-yl)octanamide (**20**) measured in Chloroform-d, 99.8 atom % D at 296 K.

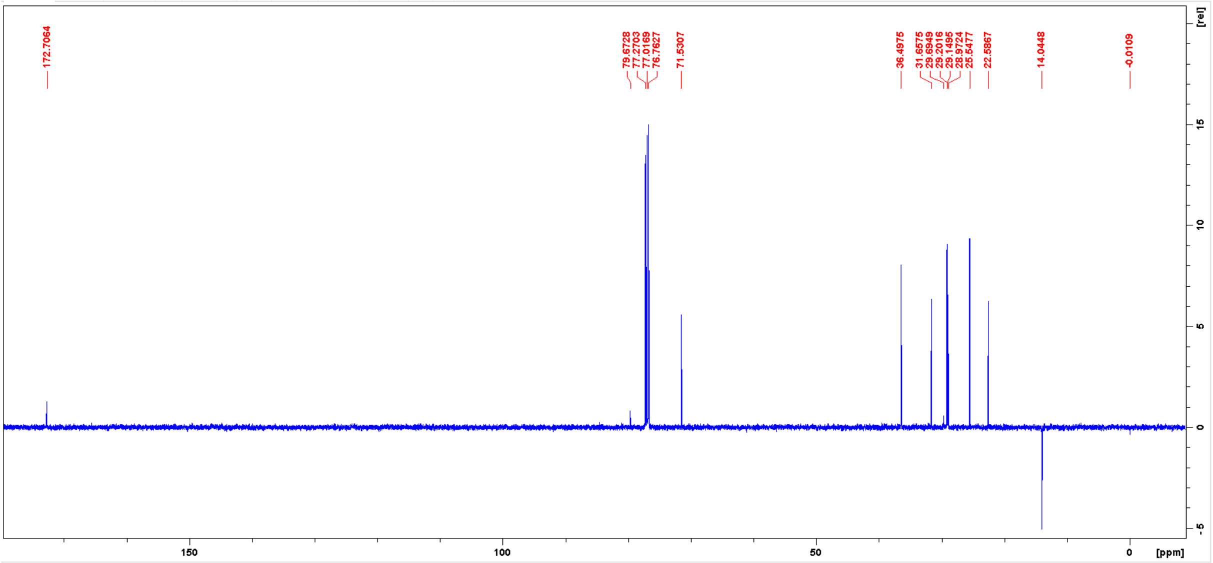

Figure S18. APT NMR spectrum of *N*-(prop-2-yn-1-yl)octanamide (**20**) measured Chloroform-d, 99.8 atom % D at 296 K.

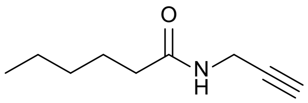

N-(prop-2-yn-1-yl)hexanamide (**21**)

The compound is obtained as a white solid in 95% yield, mp= 47.3–48.0 ⁰C (156.7 mg, 0.864 mmol).

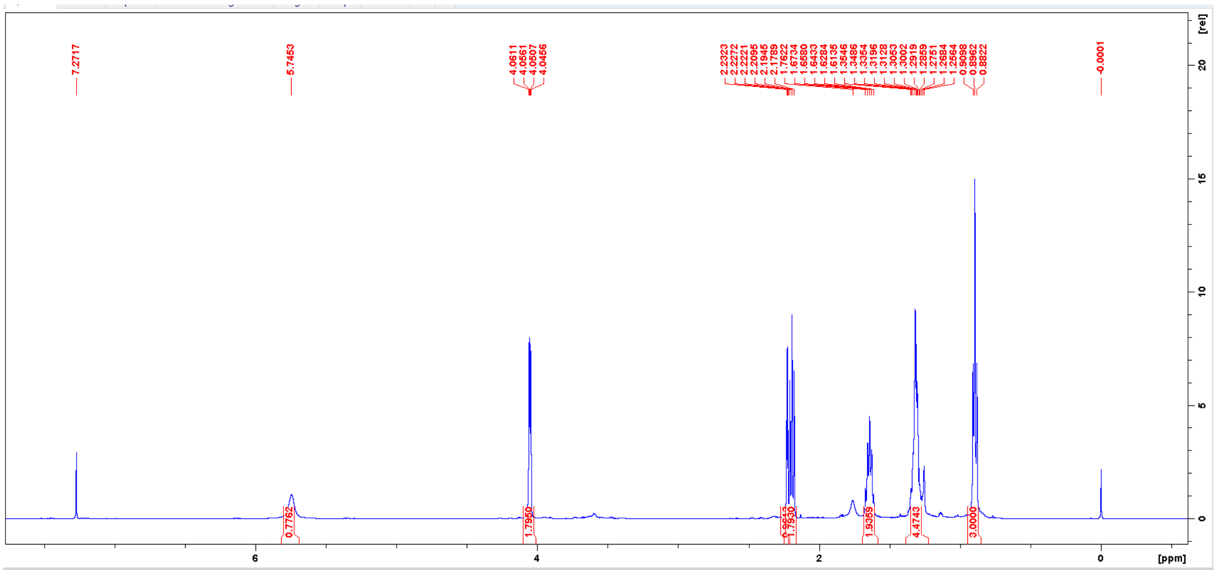

Figure S19. ^{1}H NMR spectrum of *N*-(prop-2-yn-1-yl)hexanamide (**21**) measured in Chloroform-d, 99.8 atom % D at 296 K.

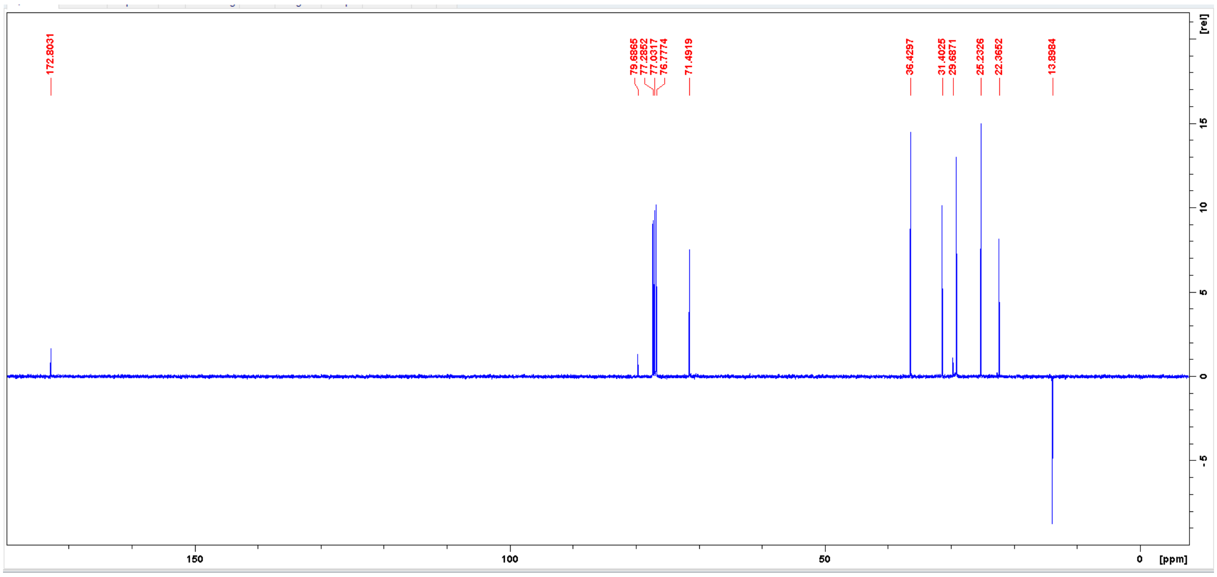

Figure S20. APT NMR spectrum of *N*-(prop-2-yn-1-yl)hexanamide (**21**) measured Chloroform-d, 99.8 atom % D at 296 K.

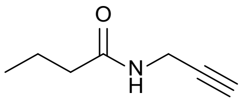

N-(prop-2-yn-1-yl)butyramide (**22**)

The compound is obtained as a yellowish solid in 90% yield, mp= 26.1–26.6 ⁰C (103.5 mg, 0.828 mmol).

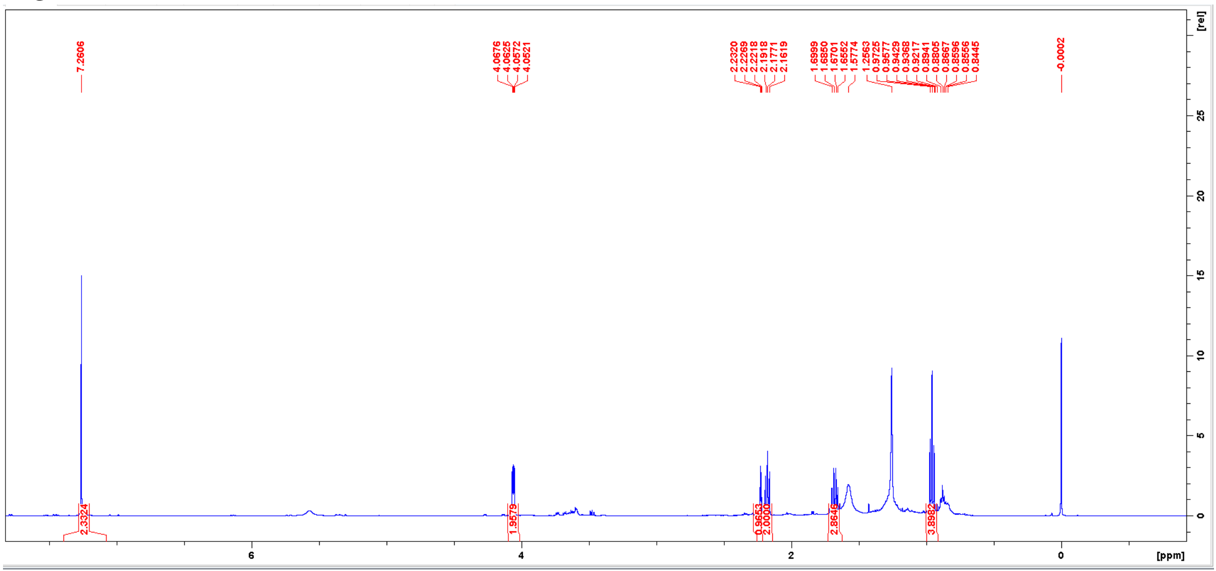

Figure S21. ^{1}H NMR spectrum of *N*-(prop-2-yn-1-yl)butyramide (**22**) measured in Chloroform-d, 99.8 atom % D at 296 K.

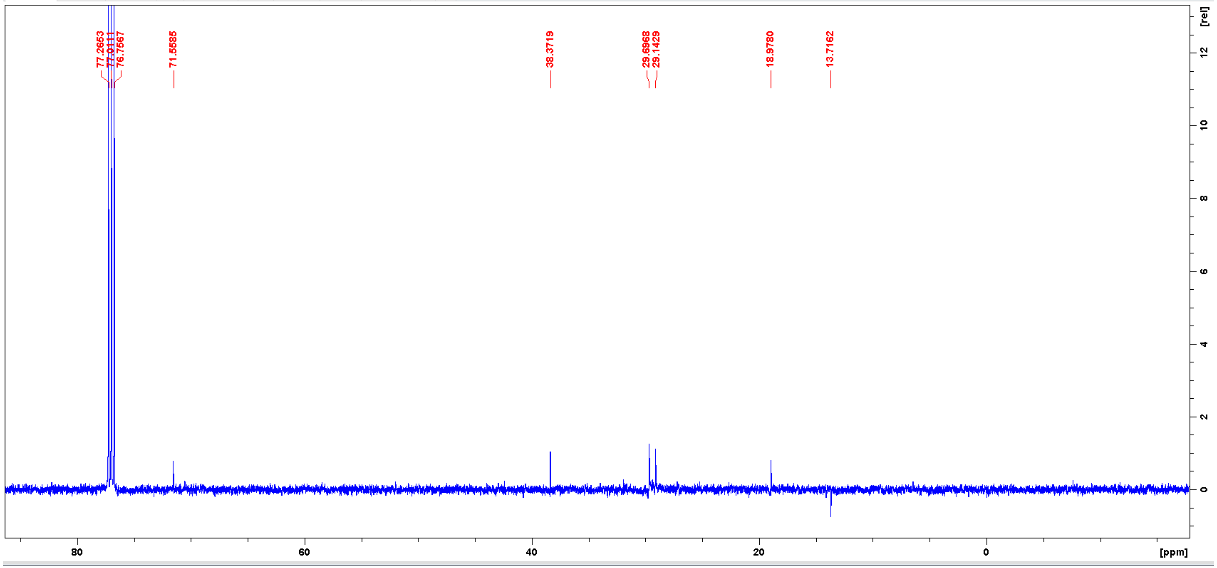

Figure S22. APT NMR spectrum of *N*-(prop-2-yn-1-yl)butyramide (**22**) measured Chloroform-d, 99.8 atom % D at 296 K.

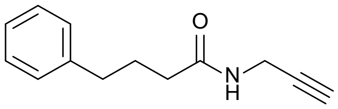

4-phenyl-*N*-(prop-2-yn-1-yl)butanamide (**23**)

The compound is obtained as a colorless oil in 93% yield (172.1 mg, 0.855 mmol).

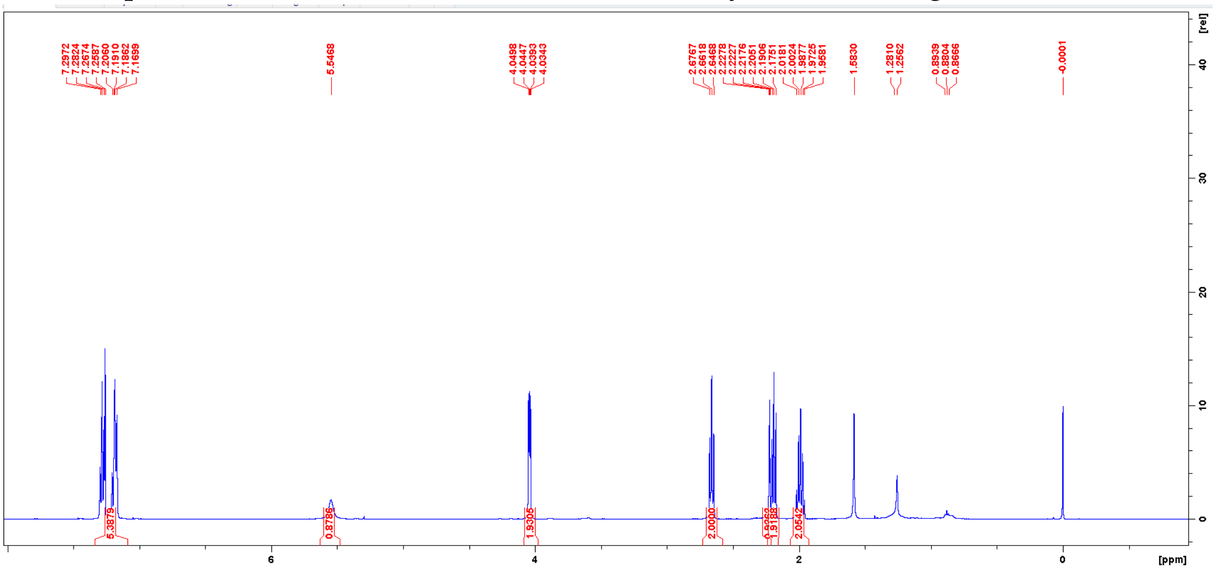

Figure S23. ^{1}H NMR spectrum of 4-phenyl-*N*-(prop-2-yn-1-yl)butanamide (**23**) measured in Chloroform-d, 99.8 atom % D at 296 K.

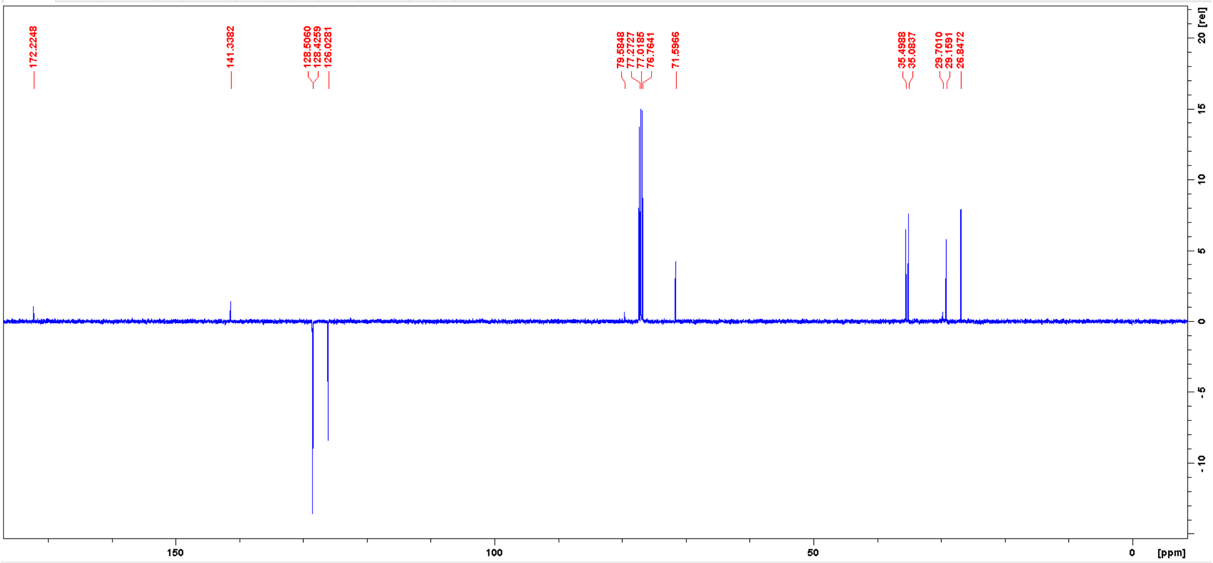

Figure S24. APT NMR spectrum of 4-phenyl-*N*-(prop-2-yn-1-yl)butanamide (**23**) measured Chloroform-d, 99.8 atom % D at 296 K.

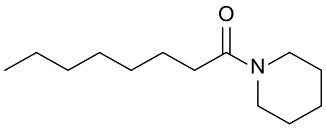

1-(piperidin-1-yl)octan-1-one (**24**)

The compound is obtained as a colorless oil in 96% yield (186.5 mg, 0.883 mmol).

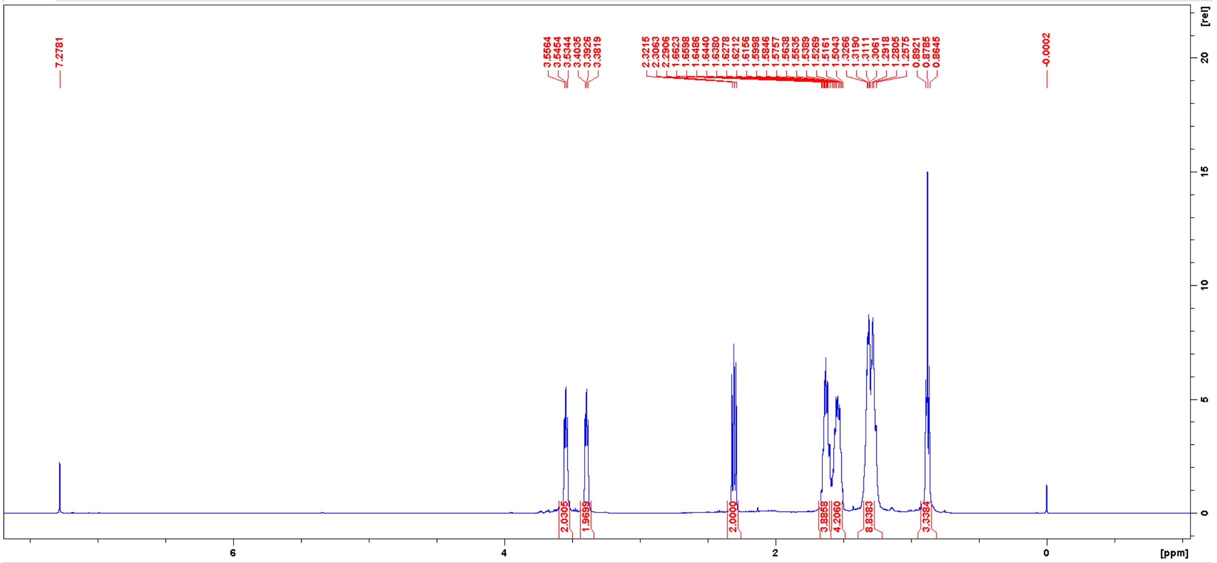

Figure S25. ^{1}H NMR spectrum of 1-(piperidin-1-yl)octan-1-one (**24**) measured in Chloroform-d, 99.8 atom % D at 296 K.

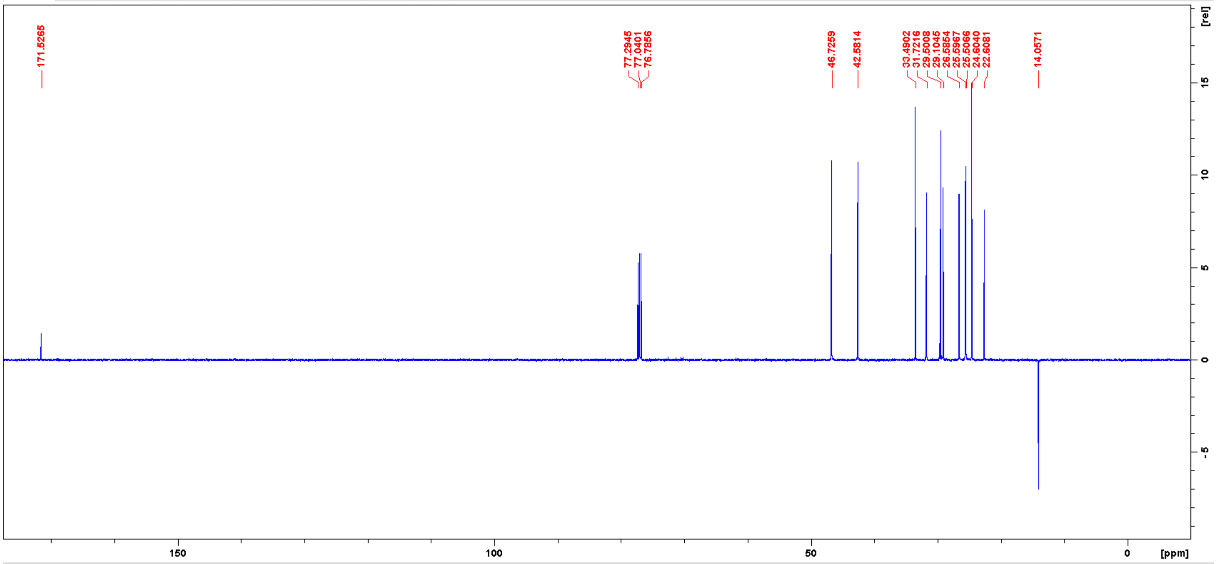

Figure S26. APT NMR spectrum of 1-(piperidin-1-yl)octan-1-one (**24**) measured Chloroform-d, 99.8 atom % D at 296 K.

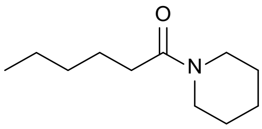

1-(piperidin-1-yl)hexan-1-one (**25**)

The compound is obtained as a yellowish oil in 90% yield (151.7 mg, 0.828 mmol).

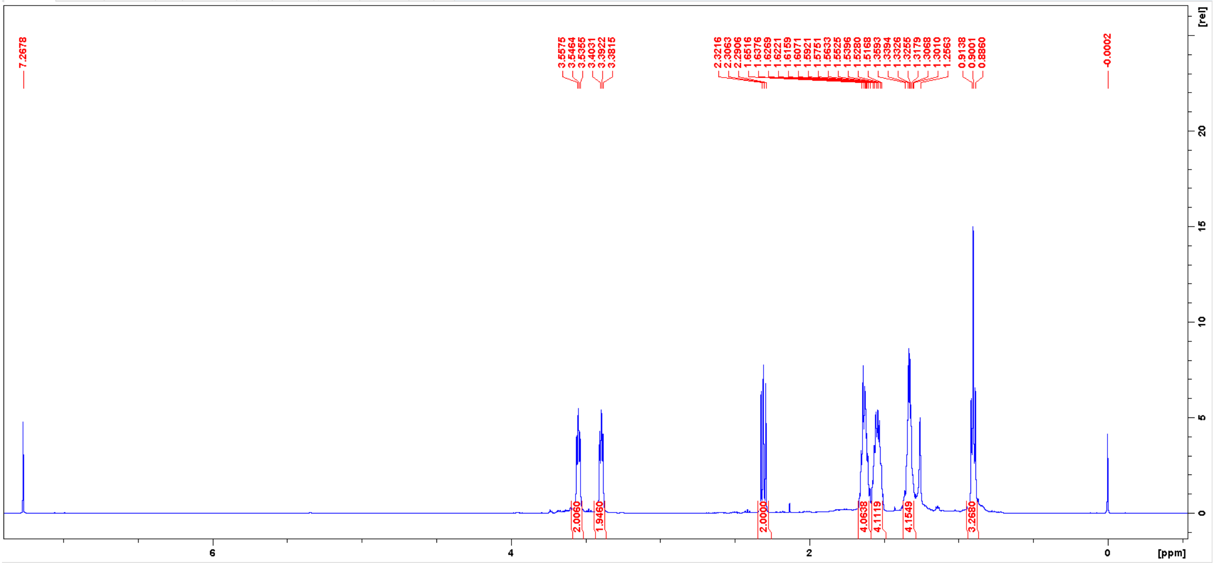

Figure S27. ^{1}H NMR spectrum of 1-(piperidin-1-yl)hexan-1-one (**25**) measured in Chloroform-d, 99.8 atom % D at 296 K.

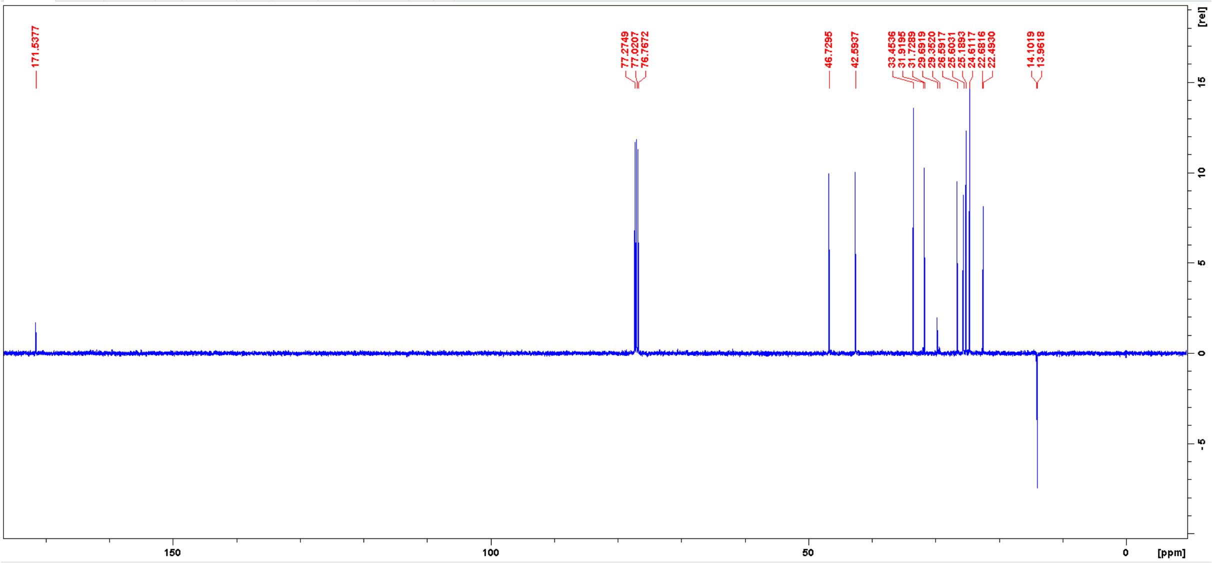

Figure S28. APT NMR spectrum of 1-(piperidin-1-yl)hexan-1-one (**25**) measured Chloroform-d, 99.8 atom % D at 296 K.

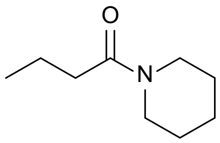

1-(piperidin-1-yl)butan-1-one (**26**)

The compound is obtained as a yellow oil in 90% yield (128.4 mg, 0.828 mmol).

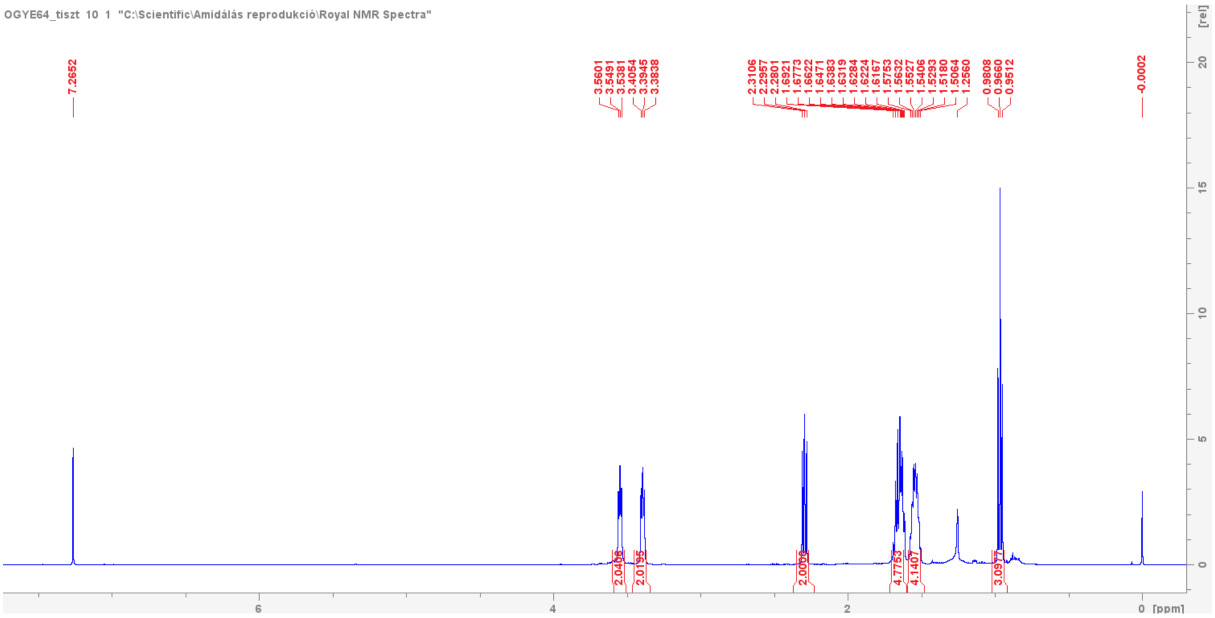

Figure S29. ^{1}H NMR spectrum of 1-(piperidin-1-yl)butan-1-one (**26**) measured in Chloroform-d, 99.8 atom % D at 296 K.

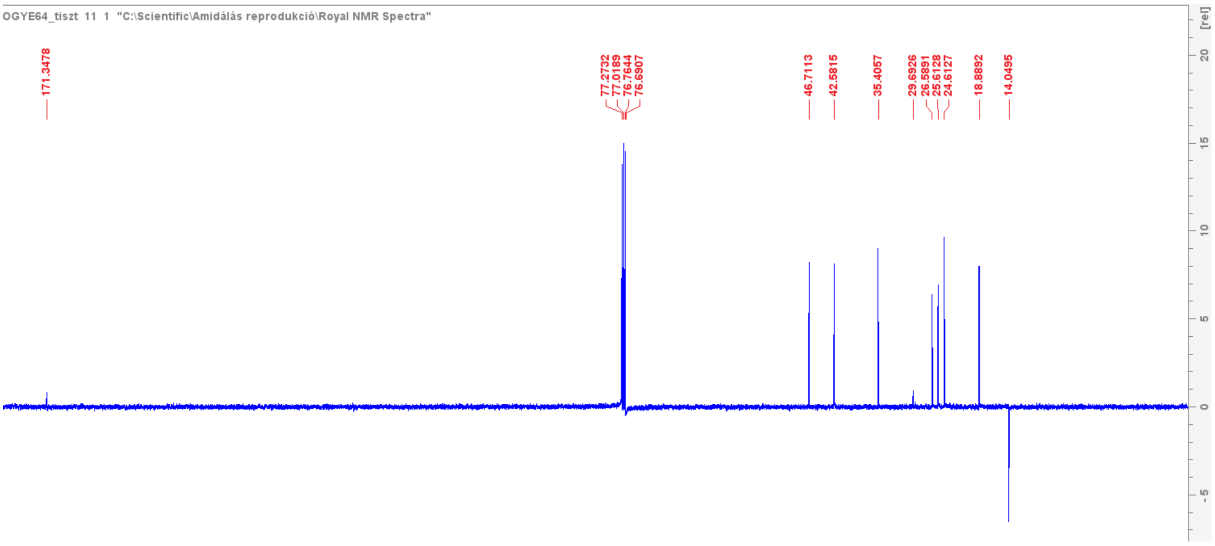

Figure S30. APT NMR spectrum of 1-(piperidin-1-yl)butan-1-one (**26**) measured in Chloroform-d, 99.8 atom % D at 296 K.

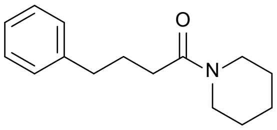

4-phenyl-1-(piperidin-1-yl)butan-1-one (**27**)

The compound is obtained as a white solid in 95% yield, mp= 153.9–154.4 °C (202.1 mg, 0.874 mmol).

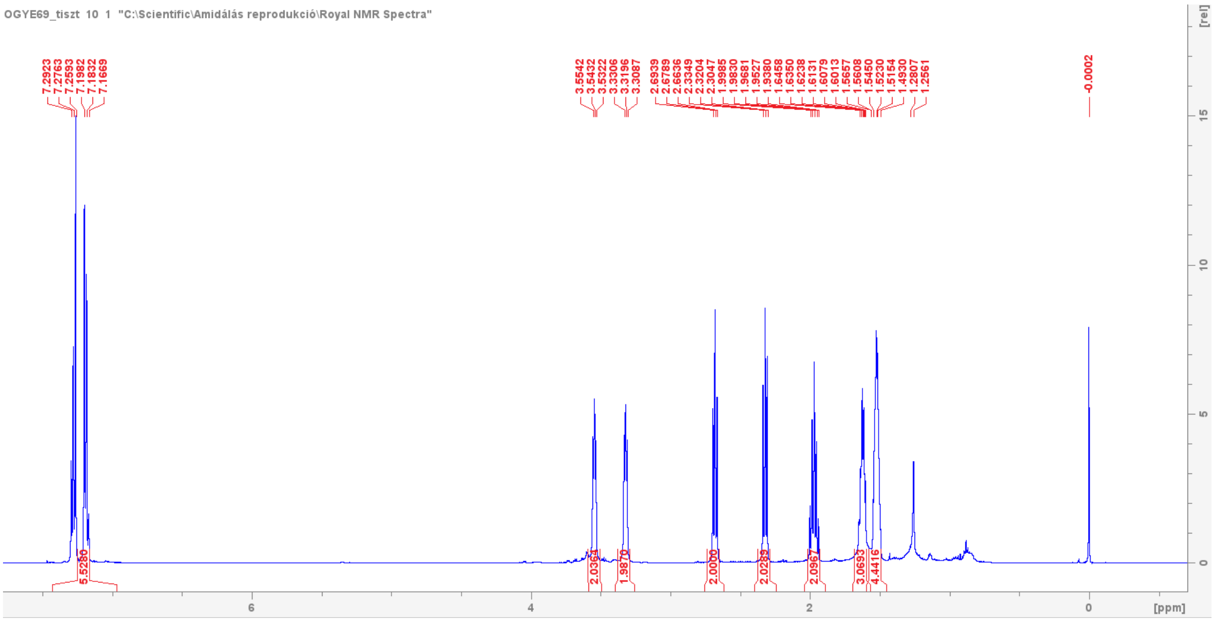

Figure S31. ^{1}H NMR spectrum of 4-phenyl-1-(piperidin-1-yl)butan-1-one (**27**) measured in Chloroform-d, 99.8 atom % D at 296 K.

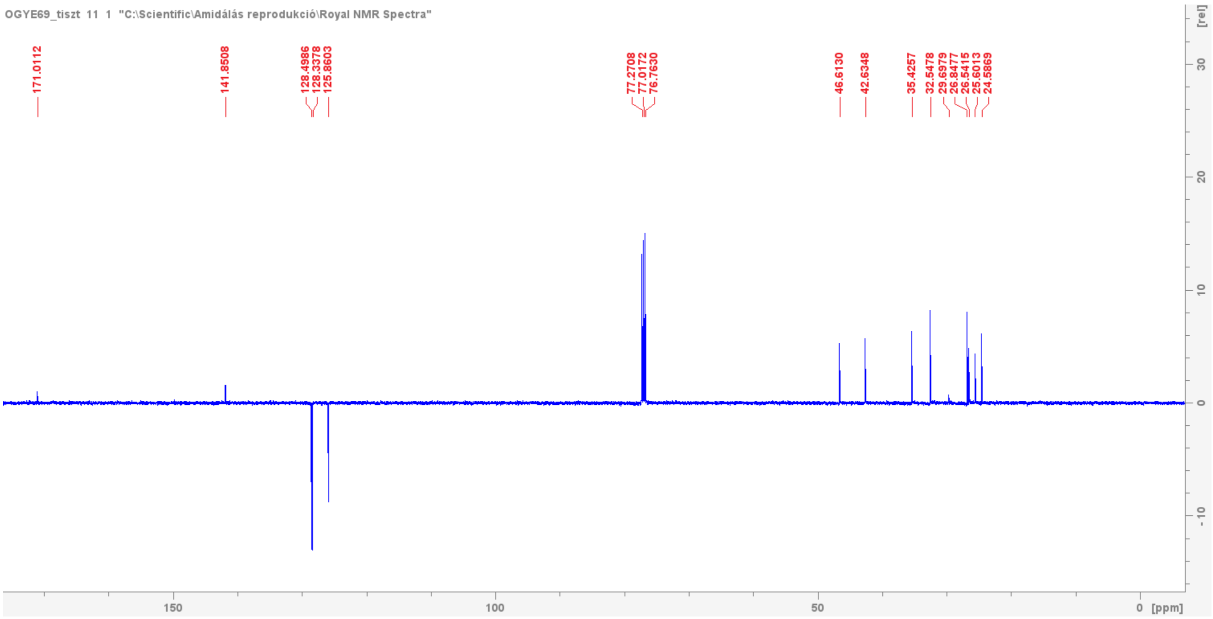

Figure S32. APT NMR spectrum of 4-phenyl-1-(piperidin-1-yl)butan-1-one (**27**) measured in Chloroform-d, 99.8 atom % D at 296 K.

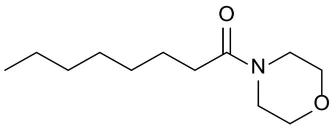

1-morpholinooctan-1-one (**28**)

The compound is obtained as a colorless oil in 96% yield (188.4 mg, 0.883 mmol).

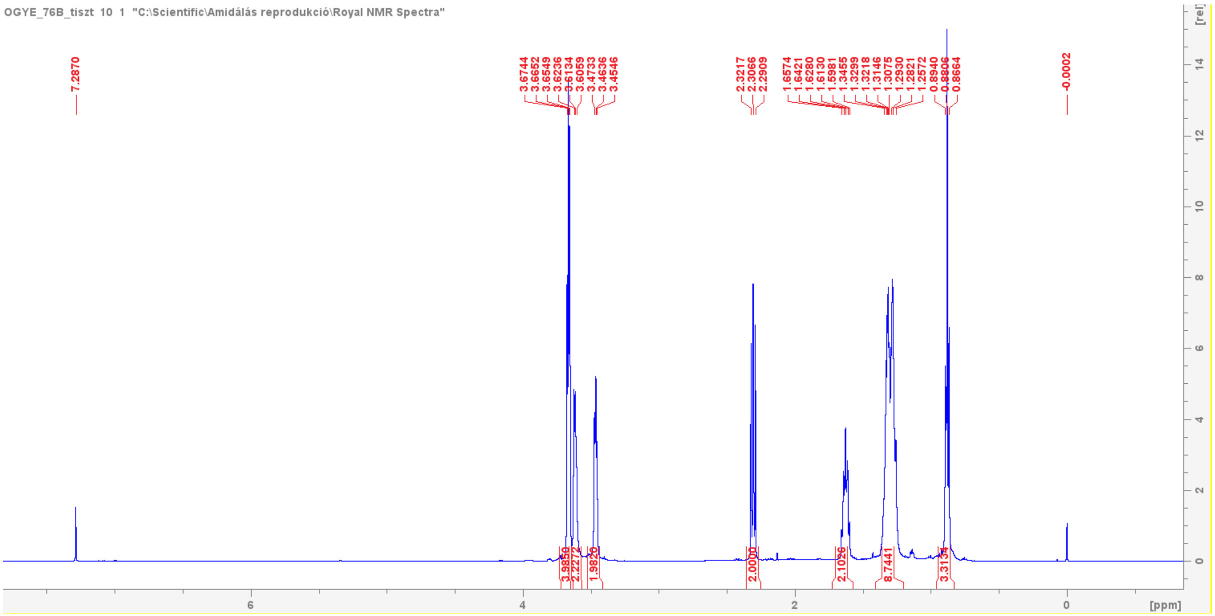

Figure S33. ^{1}H NMR spectrum of 1-morpholinooctan-1-one (**28**) measured in Chloroform-d, 99.8 atom % D at 296 K.

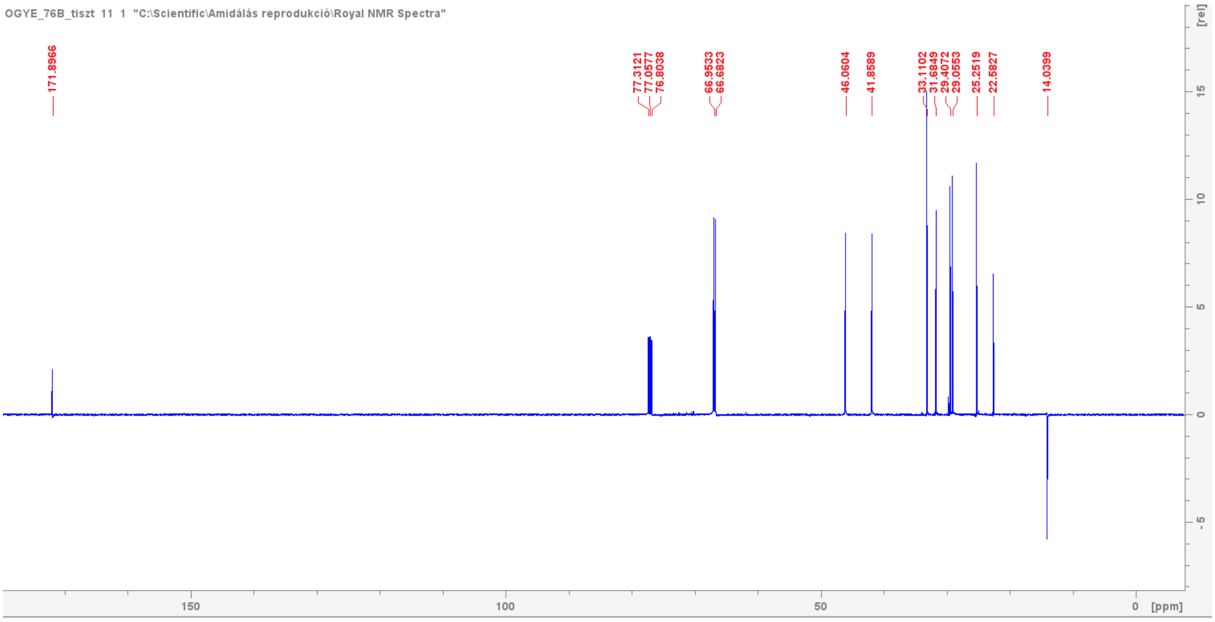

Figure S34. APT NMR spectrum of 1-morpholinooctan-1-one (**28**) measured in Chloroform-d, 99.8 atom % D at 296 K.

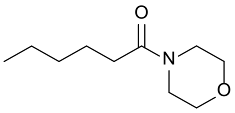

1-morpholinohexan-1-one (**29**)

The compound is obtained as a yellowish oil in 94% yield (160.2 mg, 0.864 mmol).

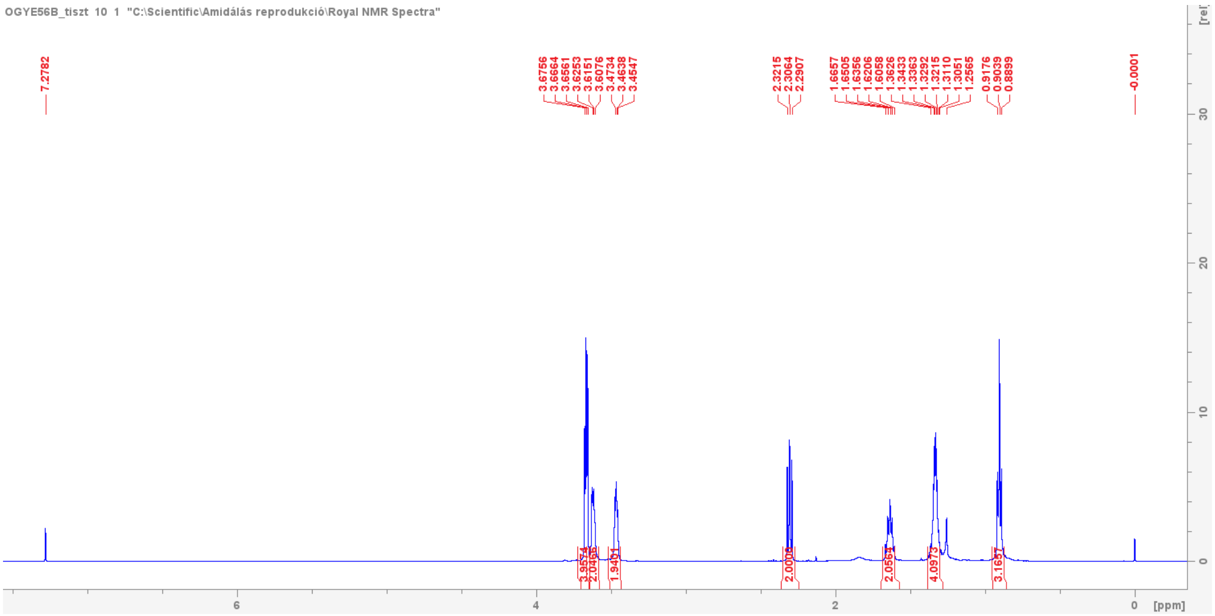

Figure S35. ^{1}H NMR spectrum of 1-morpholinohexan-1-one (**29**) measured in Chloroform-d, 99.8 atom % D at 296 K.

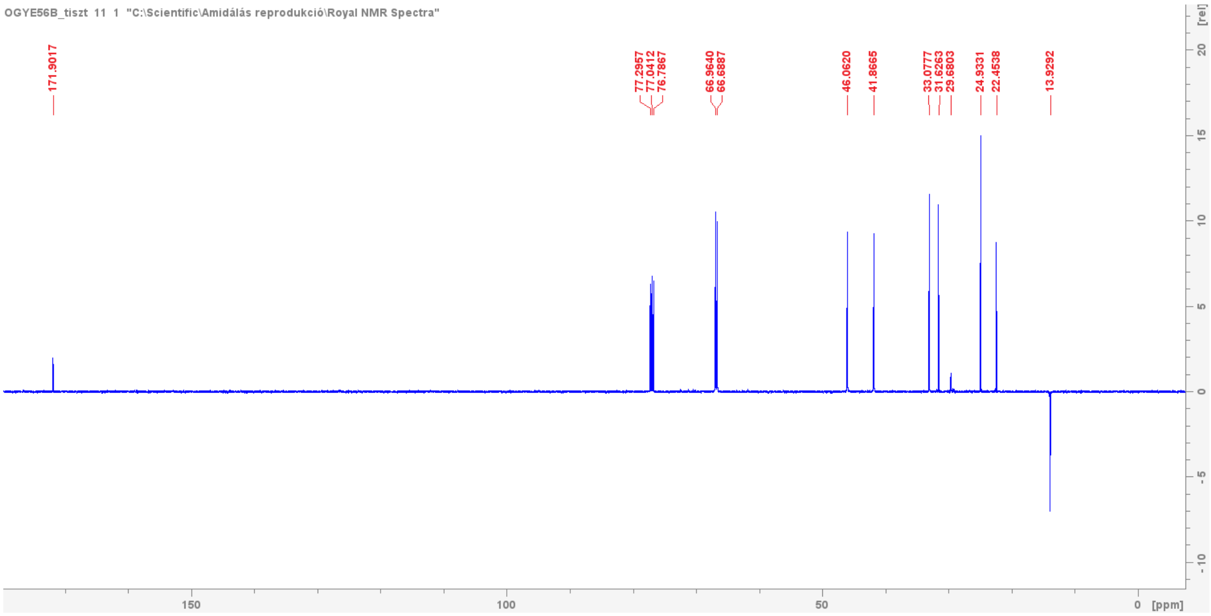

Figure S36. APT NMR spectrum of 1-morpholinohexan-1-one (**29**) measured in Chloroform-d, 99.8 atom % D at 296 K.

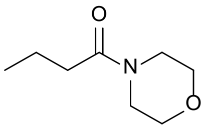

1-morpholinobutan-1-one (**30**)

The compound is obtained as a colorless oil in 91% yield (188.4 mg, 0.837 mmol).

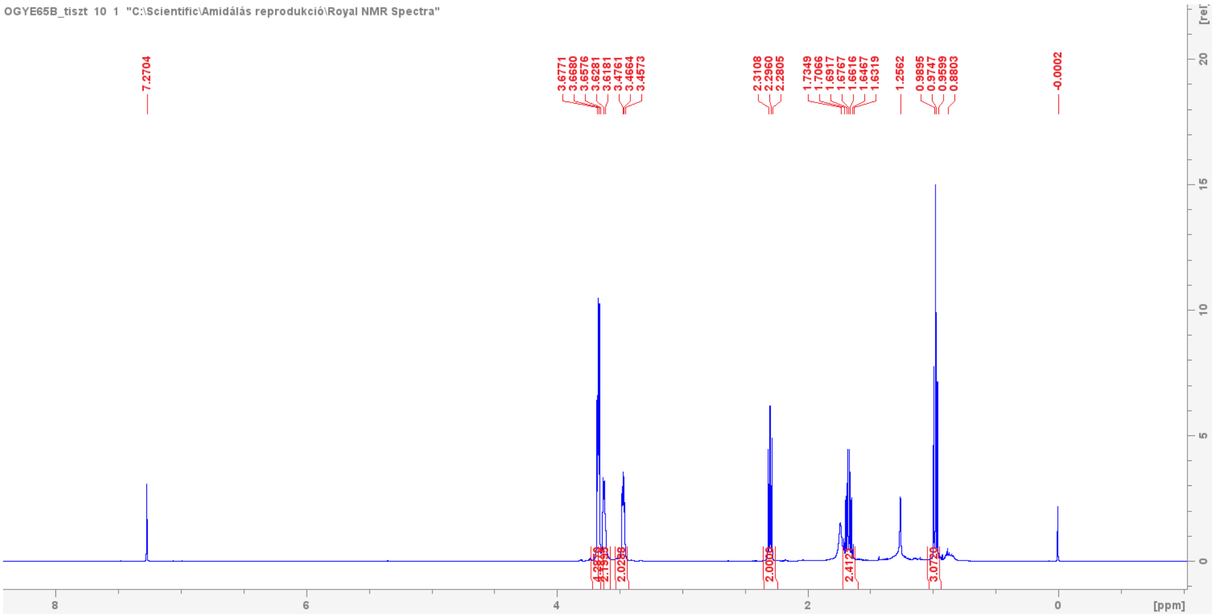

Figure S37. ^{1}H NMR spectrum of 1-morpholinobutan-1-one (**30**) measured in Chloroform-d, 99.8 atom % D at 296 K.

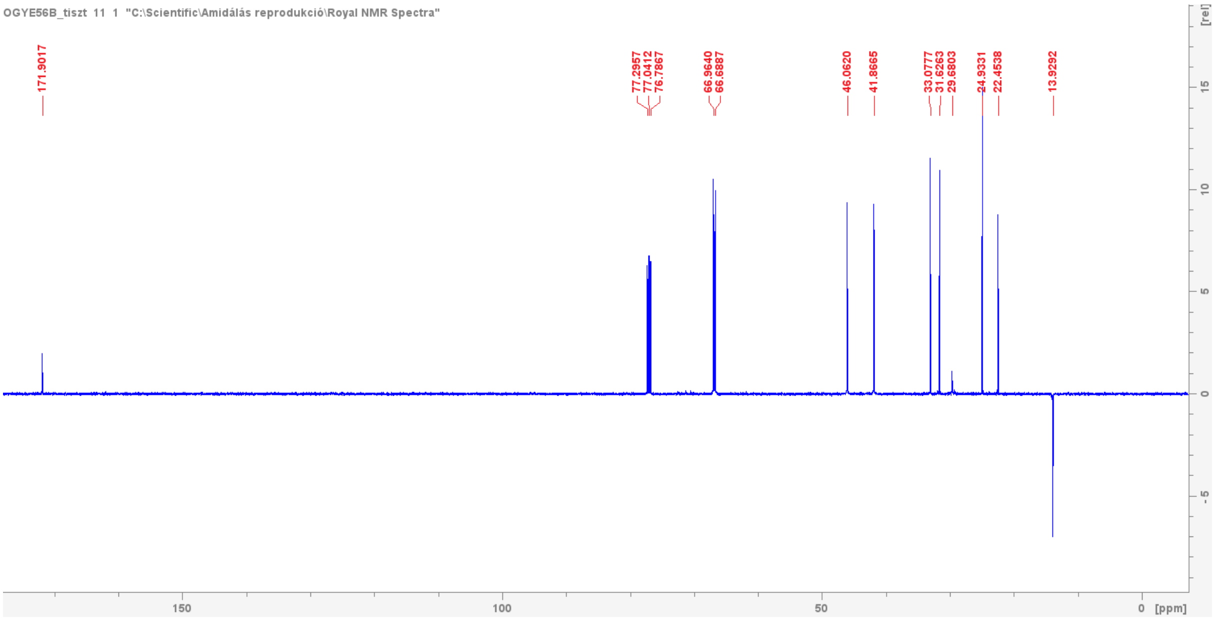

Figure S38. APT NMR spectrum of 1-morpholinobutan-1-one (**30**) measured in Chloroform-d, 99.8 atom % D at 296 K.

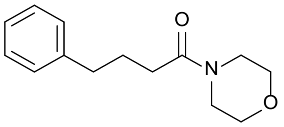

1-morpholino-4-phenylbutan-1-one (**31**)

The compound is obtained as a white solid in 92% yield, mp= 40.7–42.5 ⁰C (197.4 mg, 0.846 mmol).

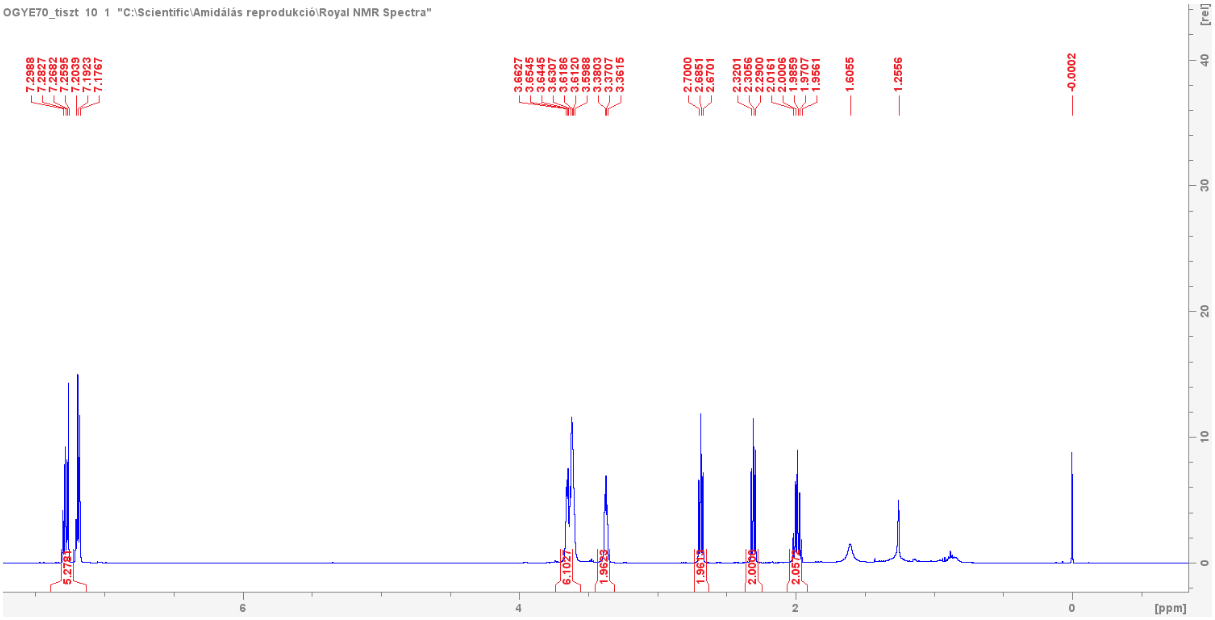

Figure S39. ^{1}H NMR spectrum of 1-morpholino-4-phenylbutan-1-one (**31**) measured in Chloroform-d, 99.8 atom % D at 296 K.

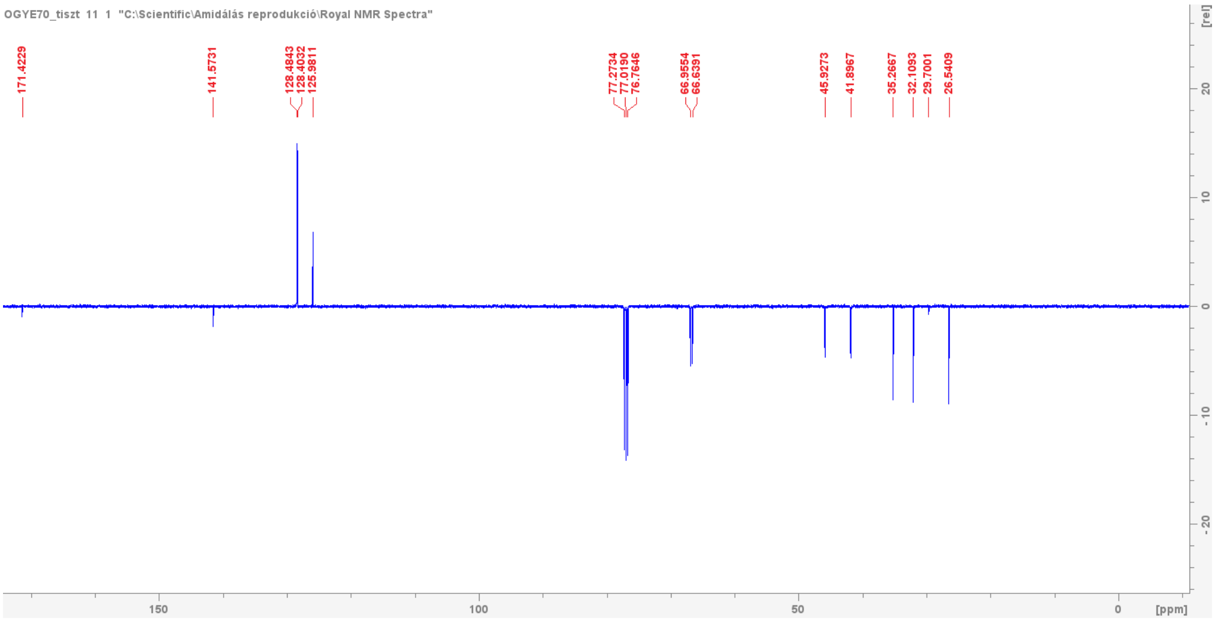

Figure S40. APT NMR spectrum of 1-morpholino-4-phenylbutan-1-one (**31**) measured in Chloroform-d, 99.8 atom % D at 296 K.

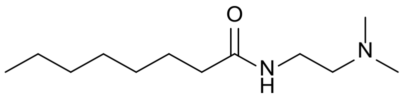

N-(2-(dimethylamino)ethyl)octanamide (**32**)

The compound is obtained as a colorless oil in 95% yield (187.3 mg, 0.874 mmol).

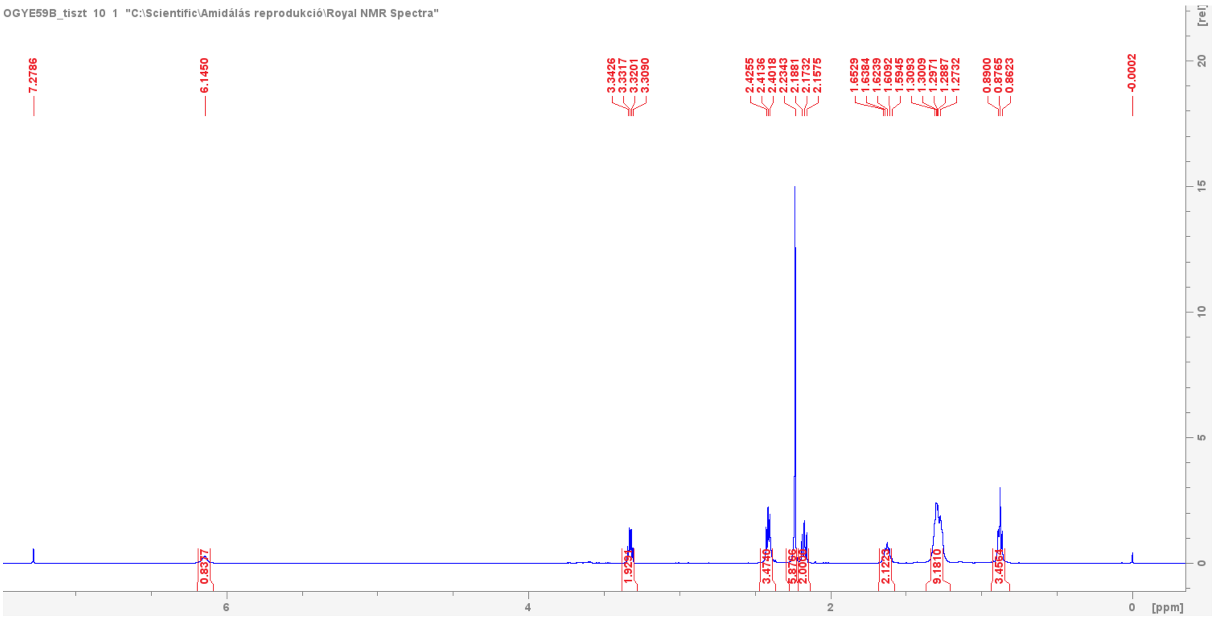

Figure S41. ^{1}H NMR spectrum of *N*-(2-(dimethylamino)ethyl)octanamide (**32**) measured in Chloroform-d, 99.8 atom % D at 296 K.

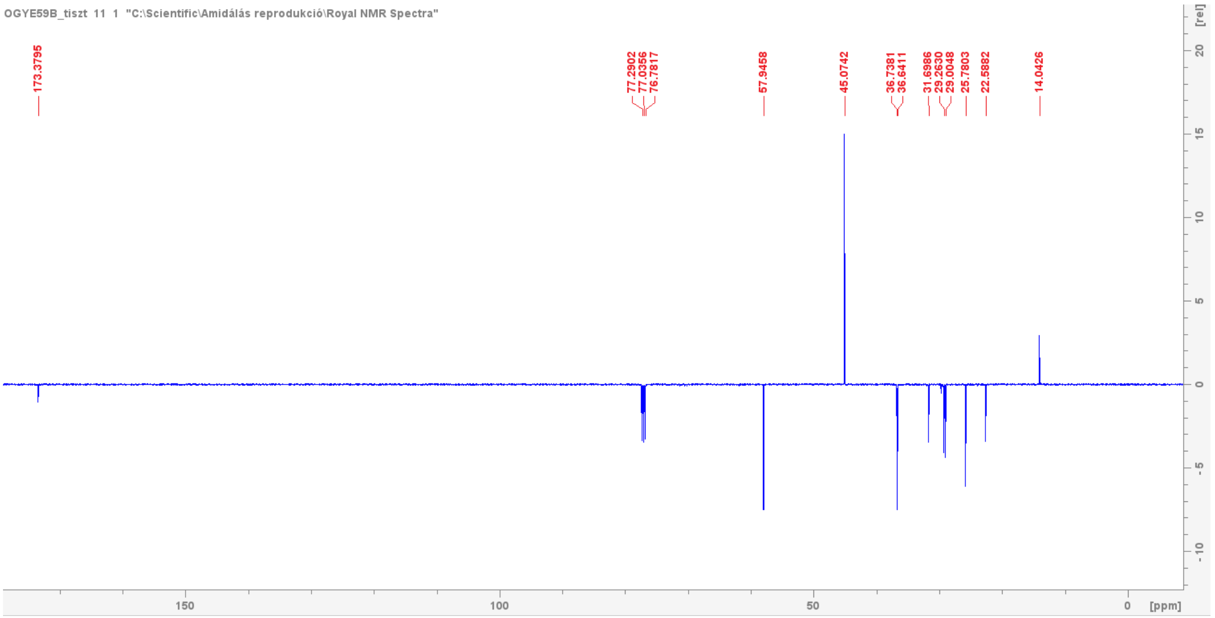

Figure S42. APT NMR spectrum of *N*-(2-(dimethylamino)ethyl)octanamide (**32**) measured in Chloroform-d, 99.8 atom % D at 296 K.

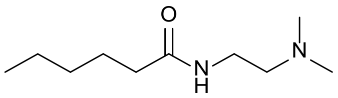

N-(2-(dimethylamino)ethyl)hexanamide (**33**)

The compound is obtained as a colorless oil in 95% yield (162.8 mg, 0.873 mmol).

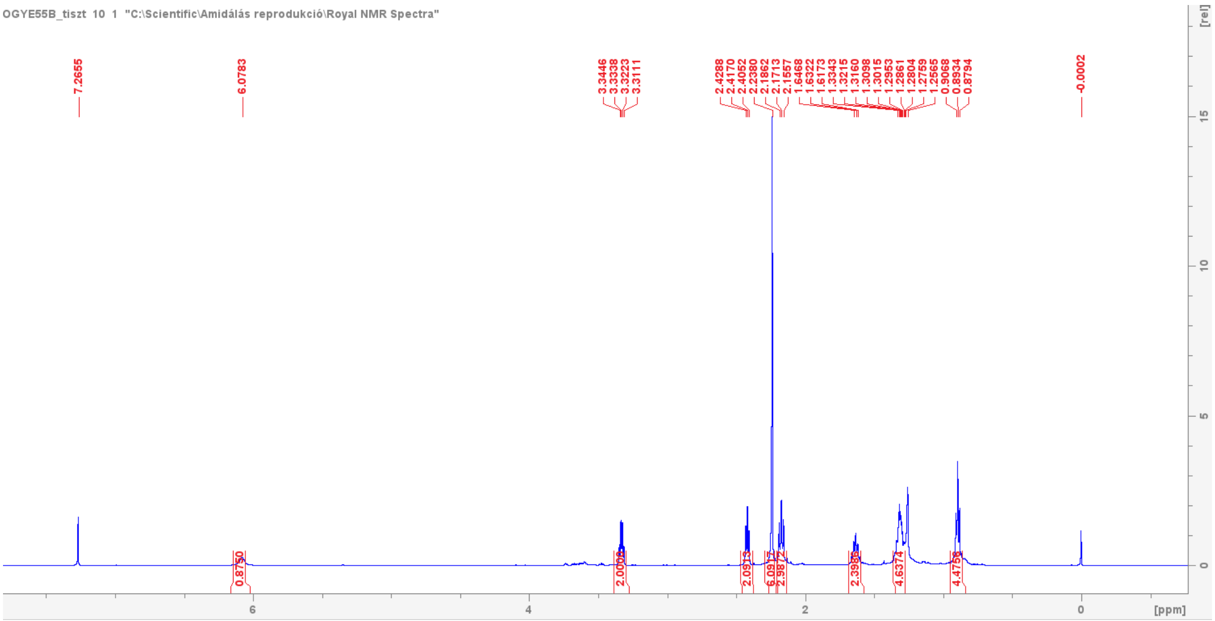

Figure S43. ^{1}H NMR spectrum of *N*-(2-(dimethylamino)ethyl)hexanamide (**33**) measured in Chloroform-d, 99.8 atom % D at 296 K.

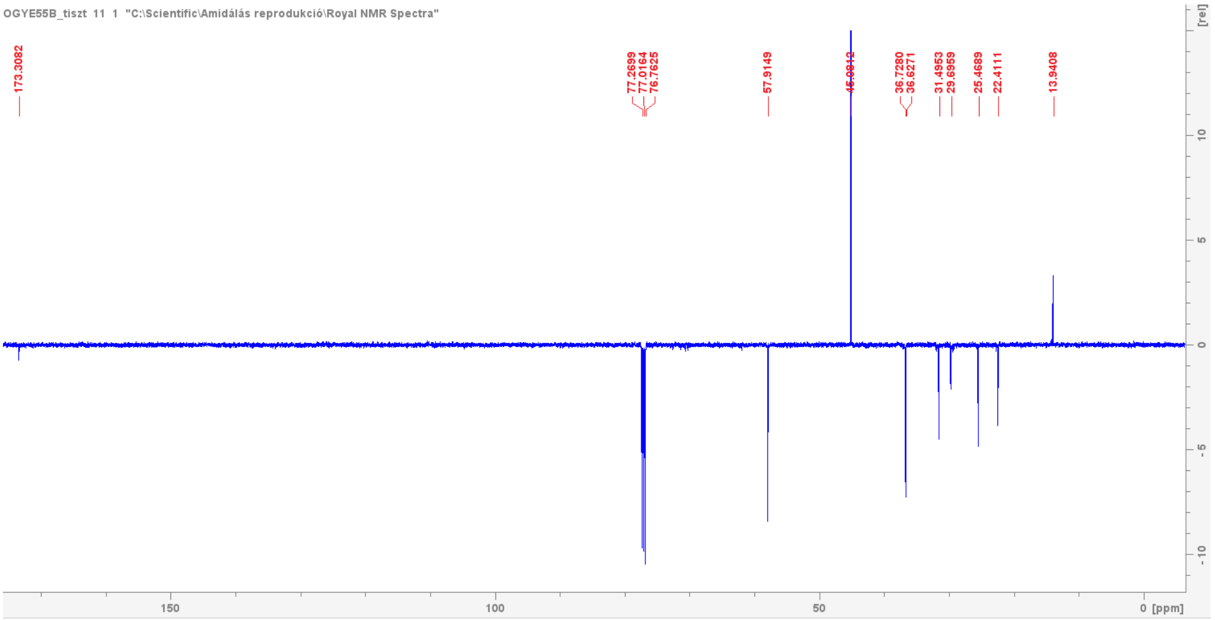

Figure S44. APT NMR spectrum of *N*-(2-(dimethylamino)ethyl)hexanamide (**33**) measured in Chloroform-d, 99.8 atom % D at 296 K.

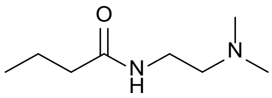

N-(2-(dimethylamino)ethyl)butyramide (**34**)

The compound is obtained as a yellowish oil in 91% yield (132.4 mg, 0.837 mmol).

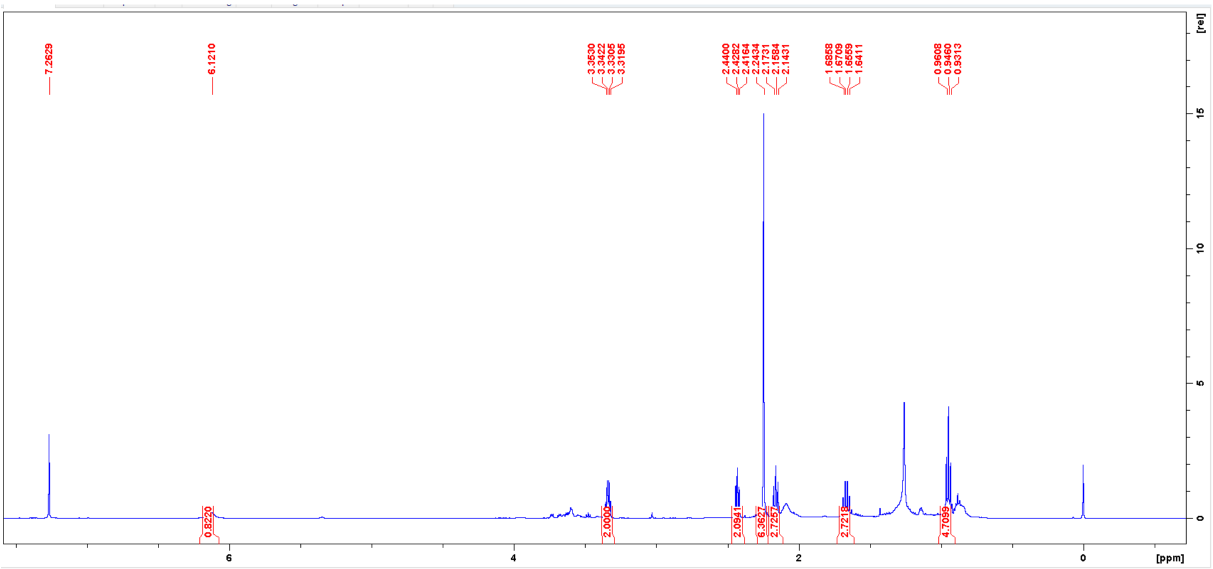

Figure S45. ^{1}H NMR spectrum of *N*-(2-(dimethylamino)ethyl)butyramide (**34**) measured in Chloroform-d, 99.8 atom % D at 296 K.

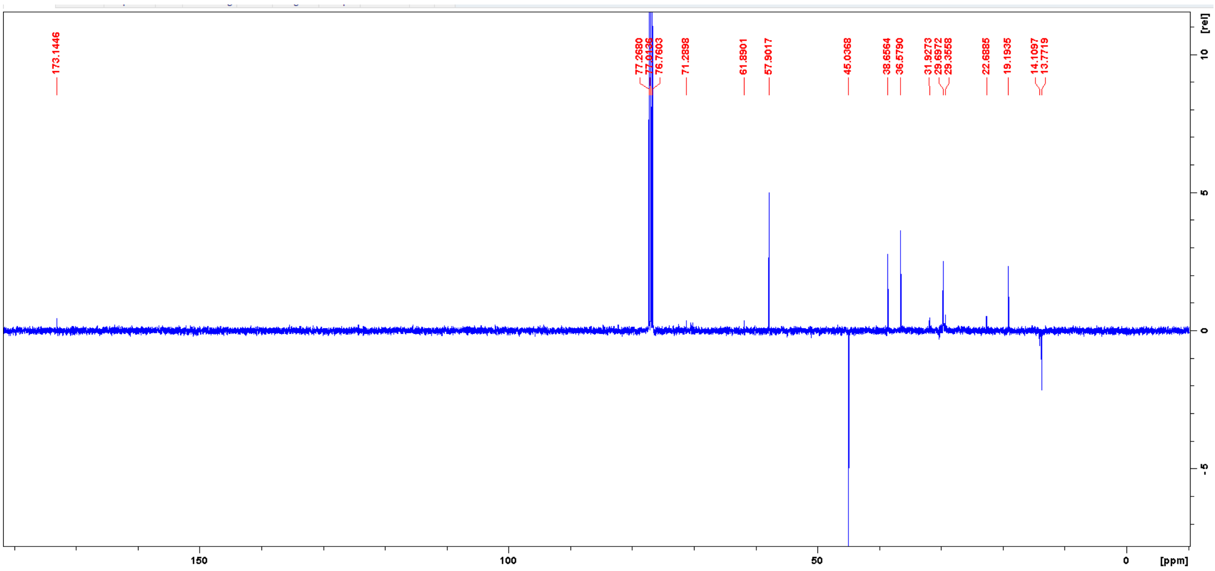

Figure S46. ^{1}H NMR spectrum of *N*-(2-(dimethylamino)ethyl)butyramide (**34**) measured in Chloroform-d, 99.8 atom % D at 296 K.

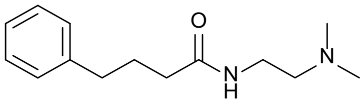

N-(2-(dimethylamino)ethyl)-4-phenylbutanamide (**35**)

The compound is obtained as a yellowish oil in 94% yield (202.6 mg, 0.864 mmol).

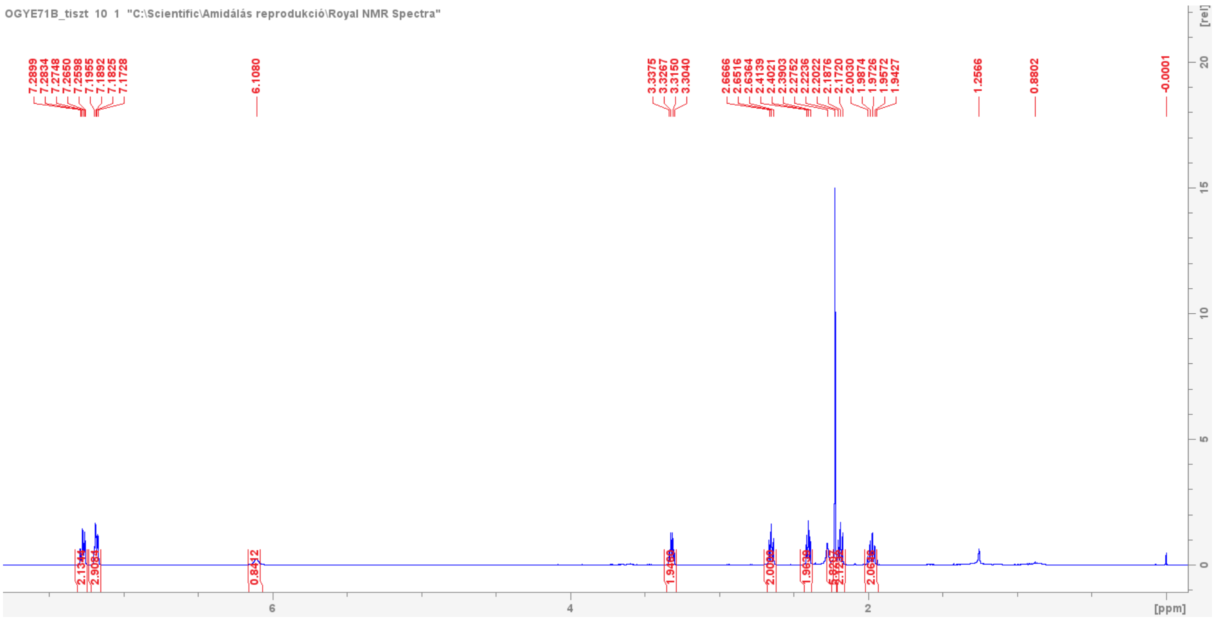

Figure S47. ^{1}H NMR spectrum of *N*-(2-(dimethylamino)ethyl)-4-phenylbutanamide (**35**) measured in Chloroform-d, 99.8 atom % D at 296 K.

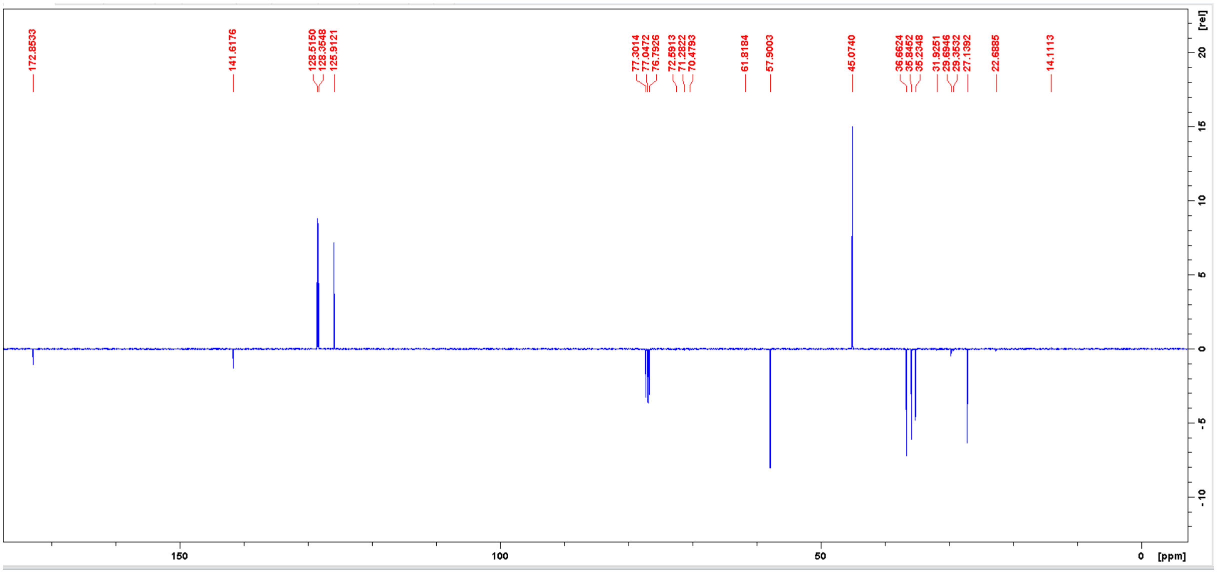

Figure S48. APT NMR spectrum of *N*-(2-(dimethylamino)ethyl)-4-phenylbutanamide (**35**) measured in Chloroform-d, 99.8 atom % D at 296 K.

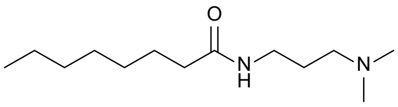

N-(3-(dimethylamino)propyl)octanamide (**36**)

The compound is obtained as a colorless oil in 94% yield (197.4 mg, 0.864 mmol).

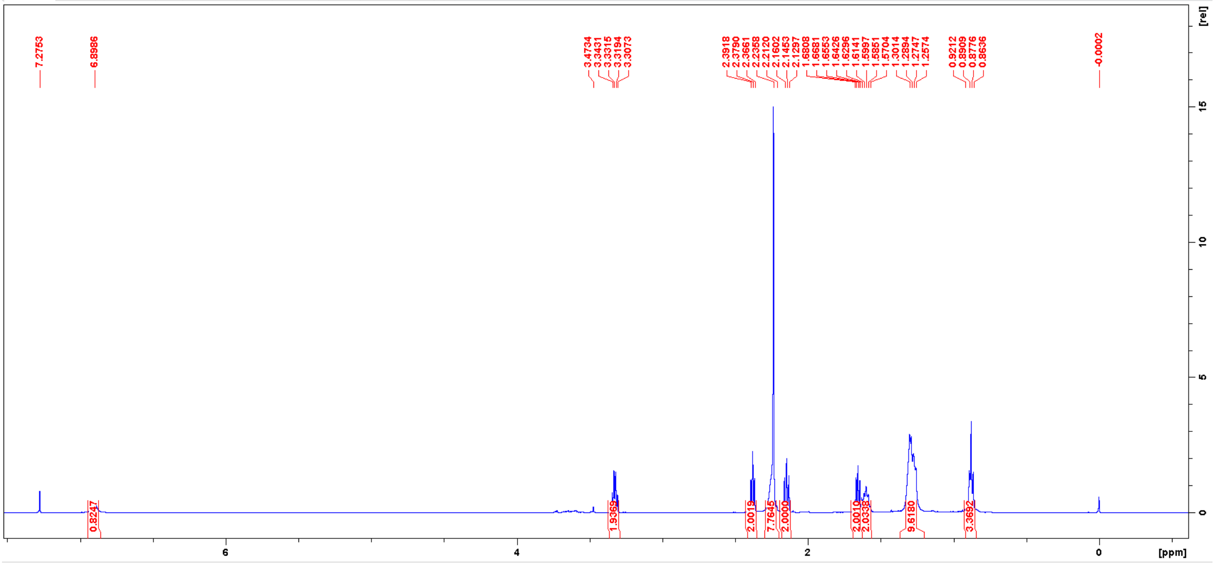

Figure S49. ^{1}H NMR spectrum of *N*-(3-(dimethylamino)propyl)octanamide (**36**) measured in Chloroform-d, 99.8 atom % D at 296 K.

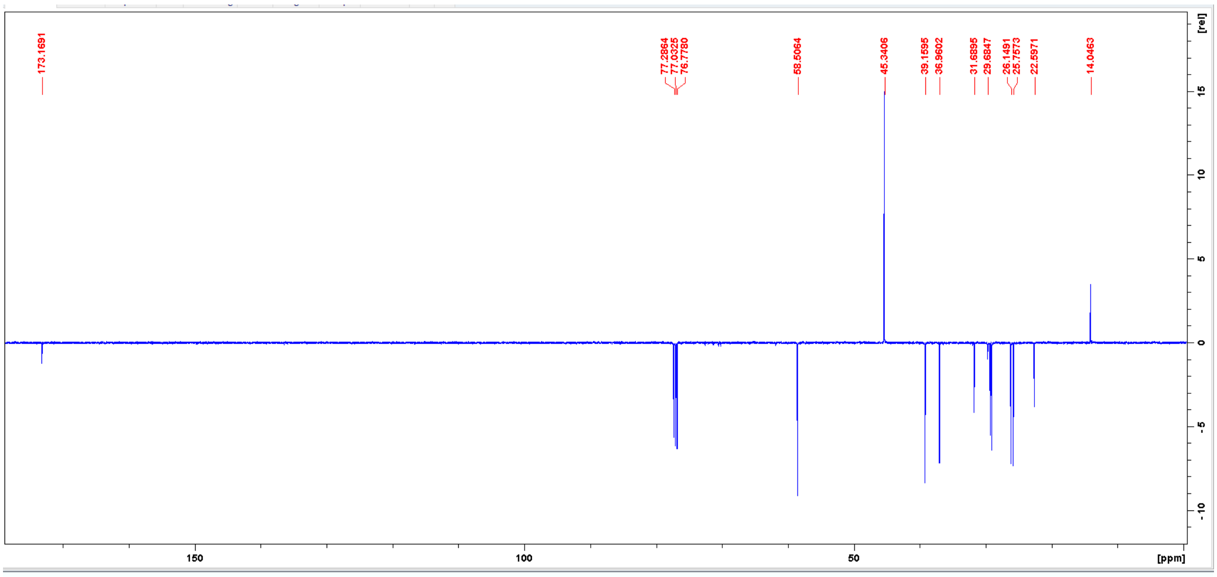

Figure S50. APT NMR spectrum of *N*-(3-(dimethylamino)propyl)octanamide (**36**) measured in Chloroform-d, 99.8 atom % D at 296 K.

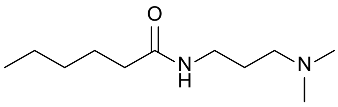

N-(3-(dimethylamino)propyl)hexanamide (**37**)

The compound is obtained as a yellowish oil in 95% yield (175.0 mg, 0.874 mmol).

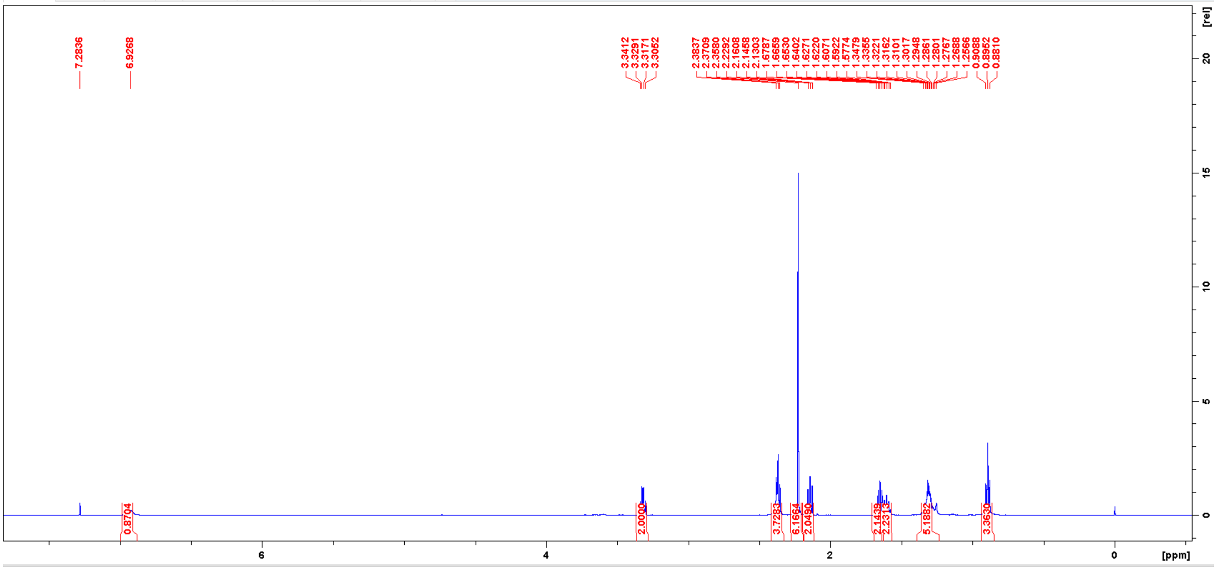

Figure S51. ^{1}H NMR spectrum of *N*-(3-(dimethylamino)propyl)hexanamide (**37**) measured in Chloroform-d, 99.8 atom % D at 296 K.

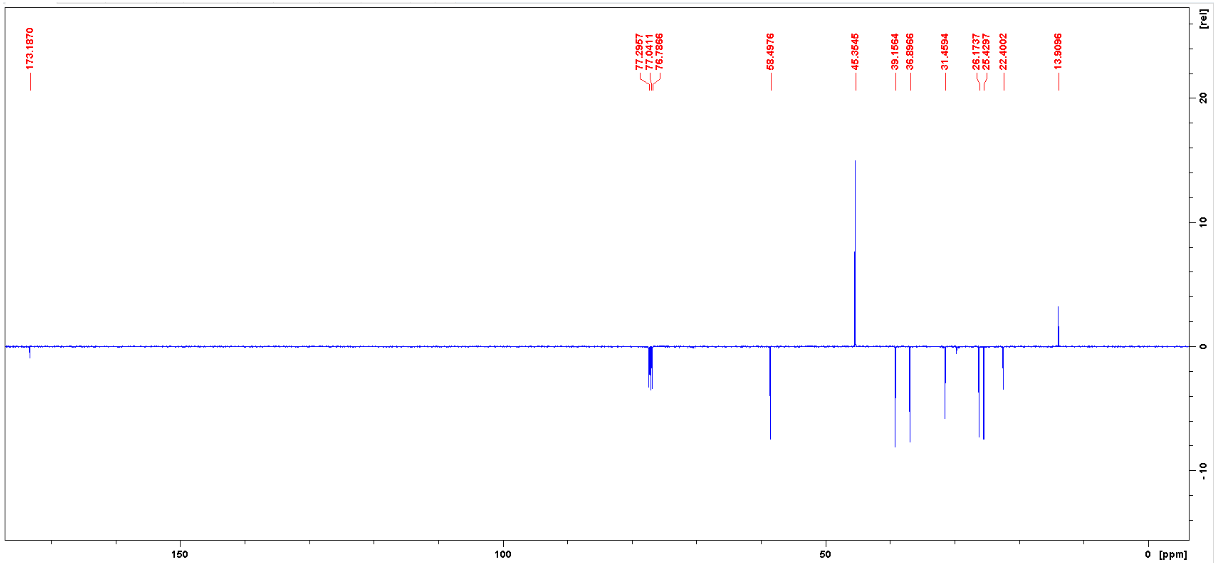

Figure S52. APT NMR spectrum of *N*-(3-(dimethylamino)propyl)hexanamide (**37**) measured in Chloroform-d, 99.8 atom % D at 296 K.

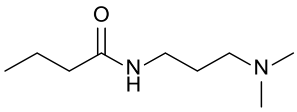

N-(3-(dimethylamino)propyl)butyramide (**38**)

The compound is obtained as a colorless oil in 90% yield (142.6 mg, 0.828 mmol).

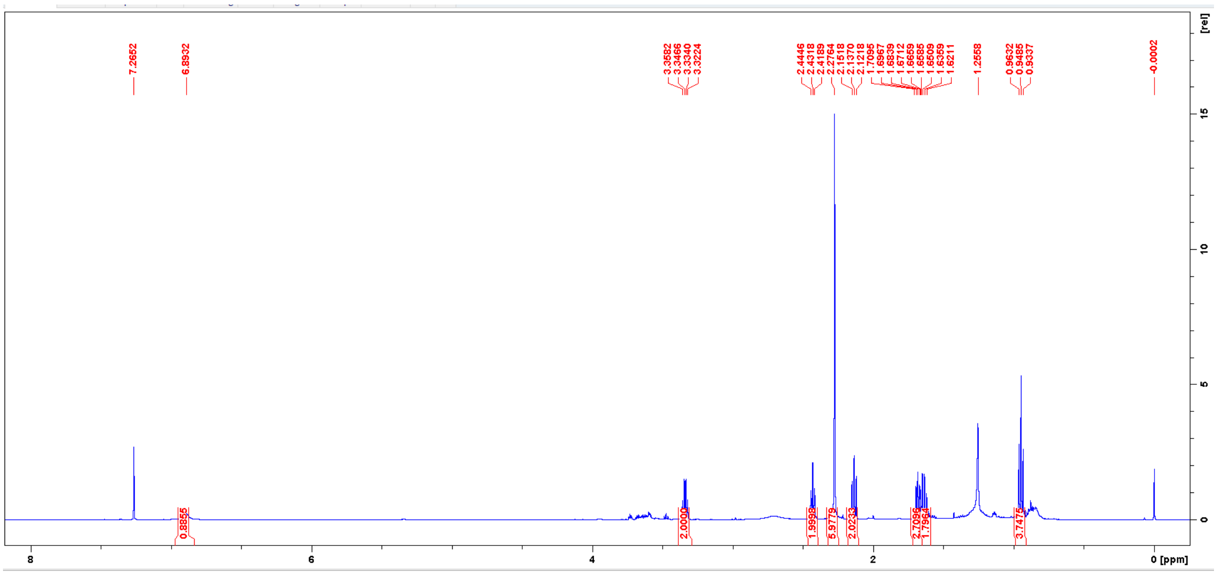

Figure S53. ^{1}H NMR spectrum of *N*-(3-(dimethylamino)propyl)butyramide (**38**) measured in Chloroform-d, 99.8 atom % D at 296 K.

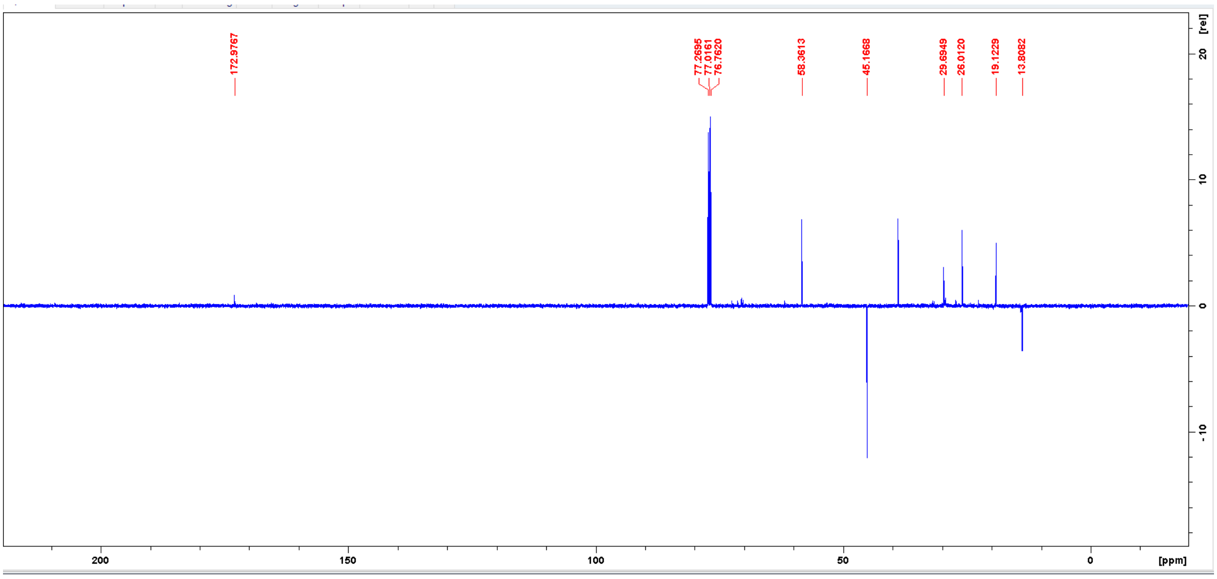

Figure S54. ^{1}H NMR spectrum of *N*-(3-(dimethylamino)propyl)butyramide (**38**) measured in Chloroform-d, 99.8 atom % D at 296 K.

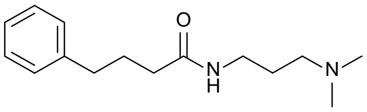

N-(3-(dimethylamino)propyl)-4-phenylbutanamide (**39**)

The compound is obtained as a yellowish oil in 95% yield (217.0 mg, 0.874 mmol).

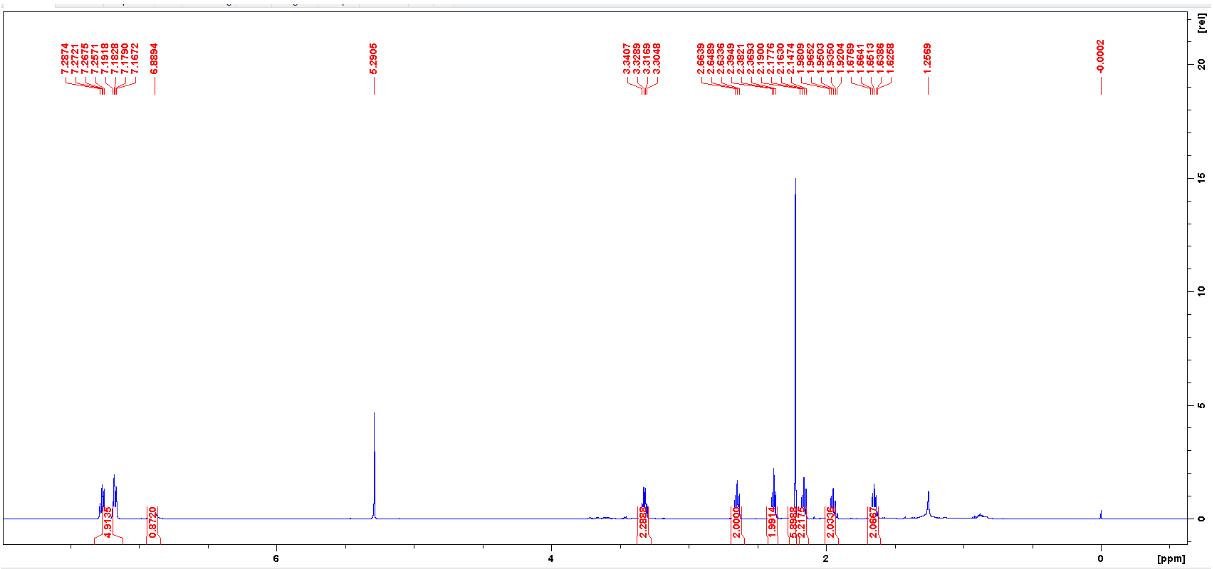

Figure S55. ^{1}H NMR spectrum of *N*-(3-(dimethylamino)propyl)-4-phenylbutanamide (**39**) measured in Chloroform-d, 99.8 atom % D at 296 K.

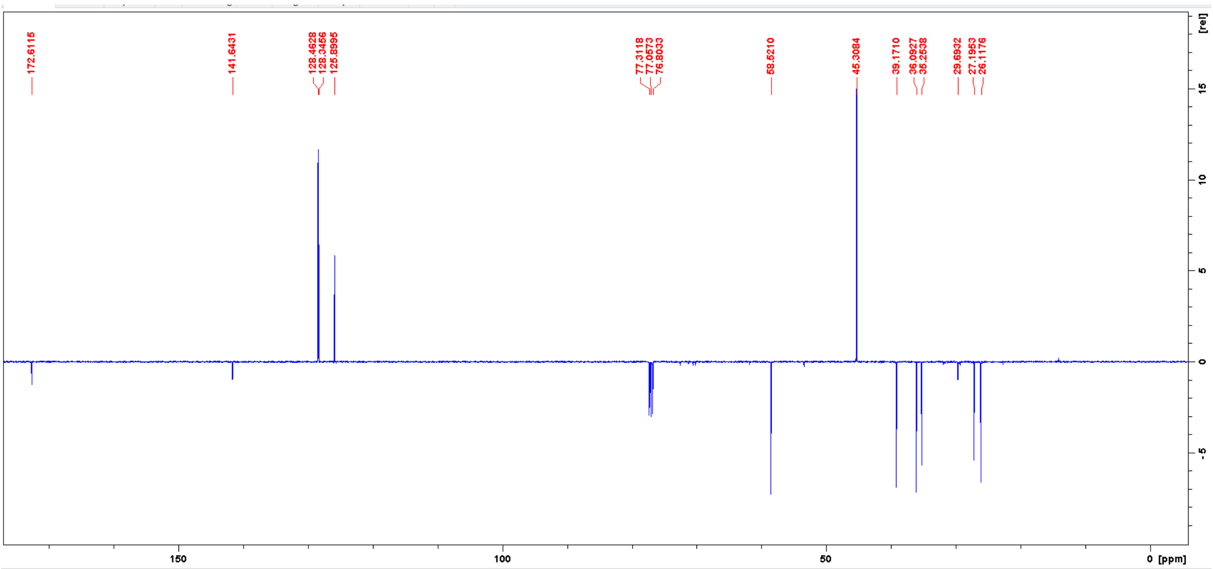

Figure S56. ^{1}H NMR spectrum of *N*-(3-(dimethylamino)propyl)-4-phenylbutanamide (**39**) measured in Chloroform-d, 99.8 atom % D at 296 K.

2. GC-MS measurement

In a screw-capped tube are successively introduced the amine (1 equiv), the acid (1 equiv) dissolved in 1 mL toluene solvent to provide a 42 mM solution. Concentration of immobilized CALB was 50 mg/mL for the above mentioned reasons. The reaction was carried out with 50 mg 3Å size molecular sieves and also 2 µl *n*-heptadecane as an analytical standard was added in the reaction mixture. 15 µl samples were taken every 10 minutes for 30 minutes.

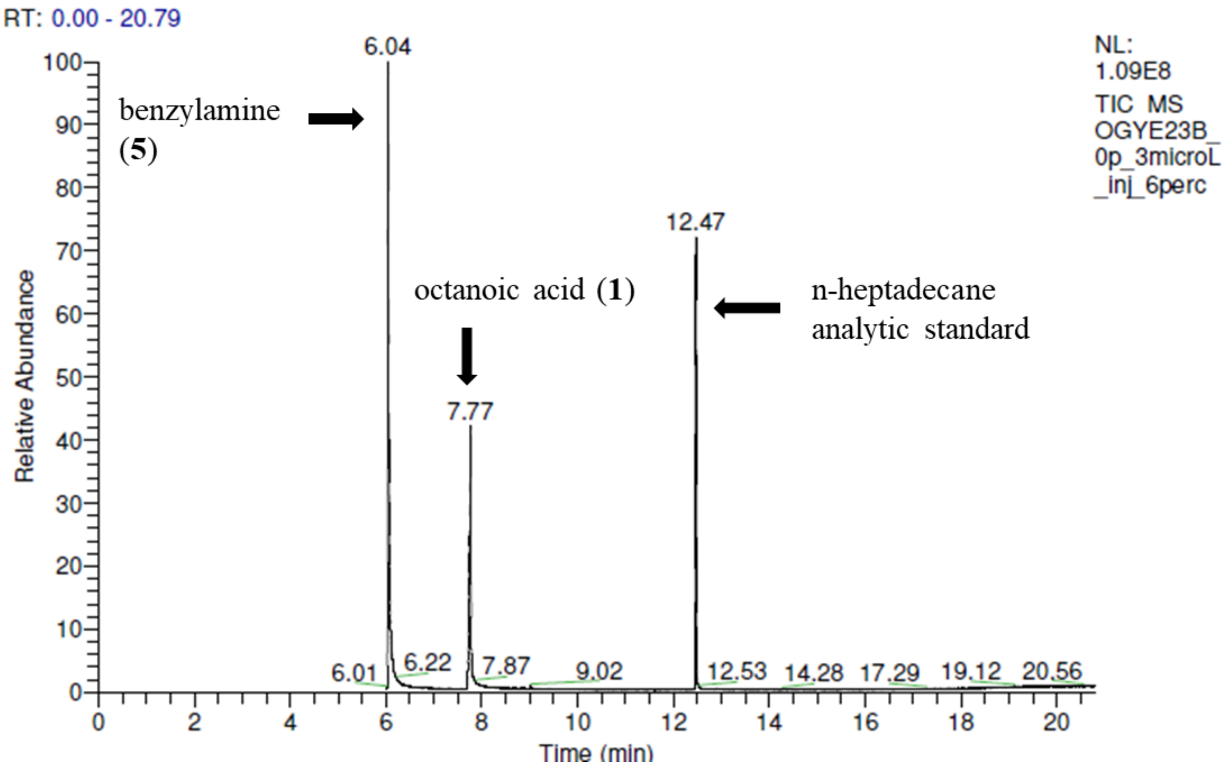

Figure S57 Total ion chromatogram of the sample of reaction mixture at the zero time by GC-MS.

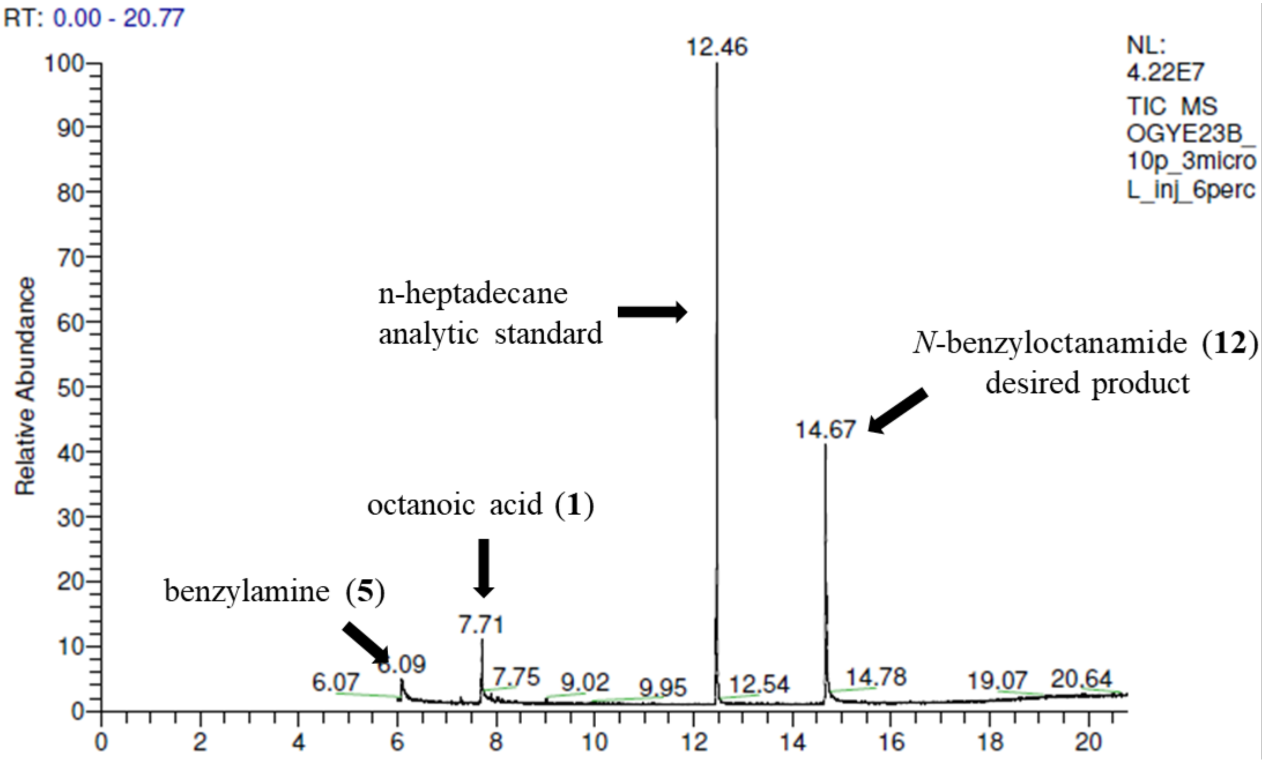

Figure S58. Total ion chromatogram of the sample of reaction mixture at the 10 minutes by GC-MS.

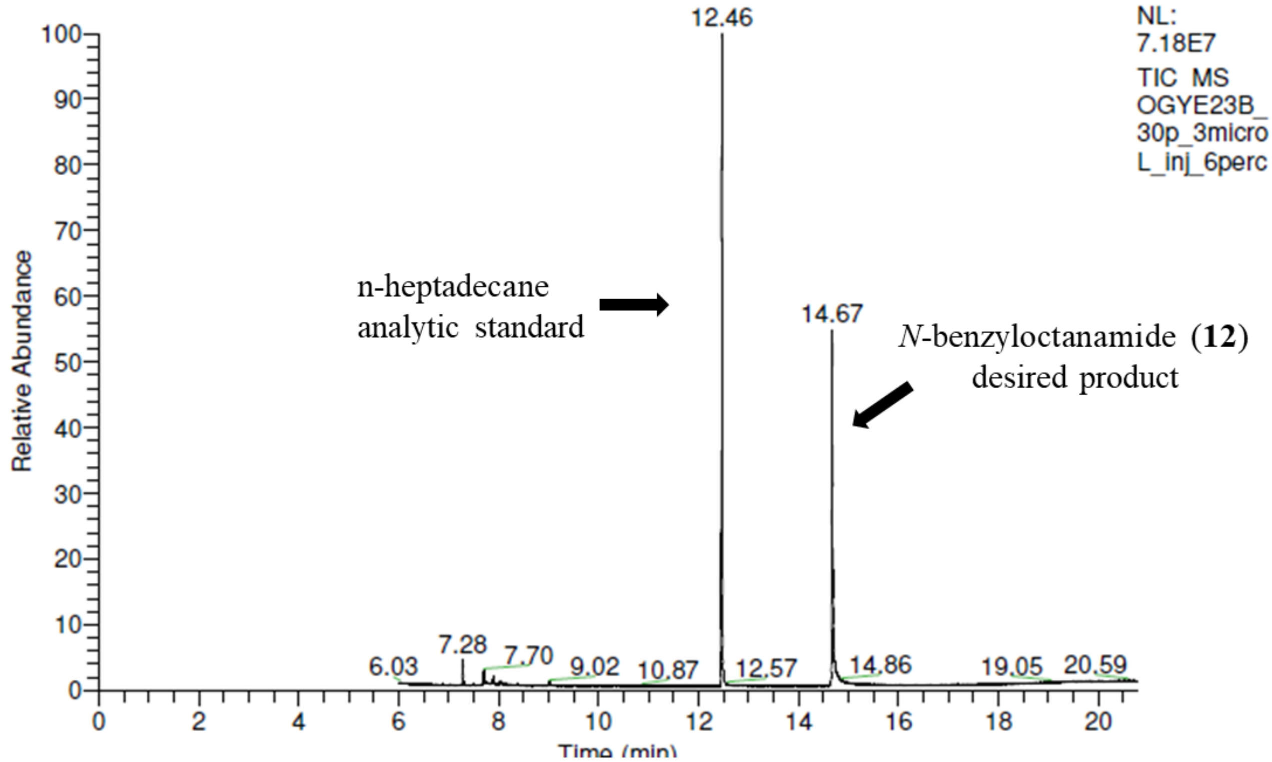

Figure S59. Total ion chromatogram of the sample of reaction mixture at the 30 minutes by GC-MS.

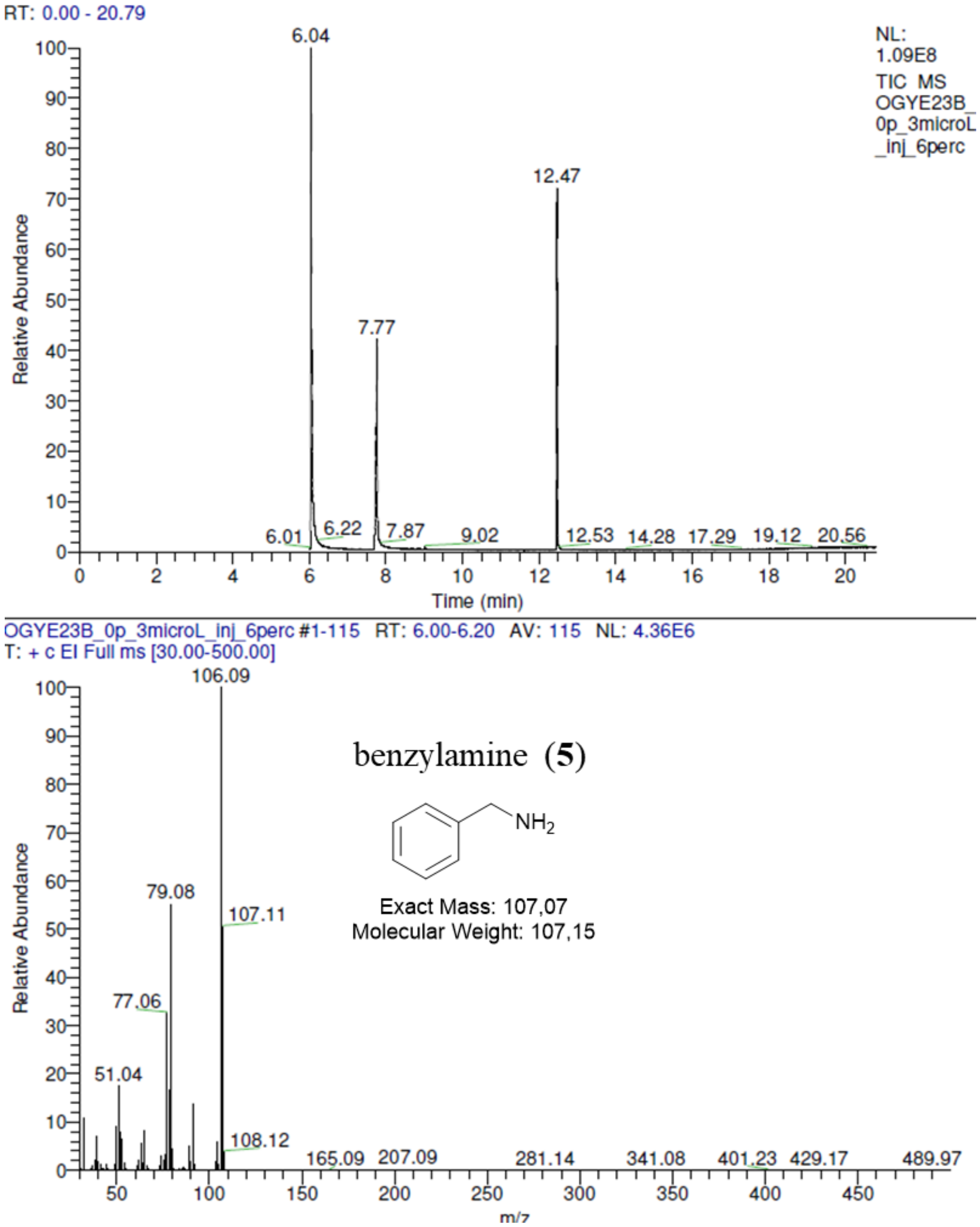

Figure S60. GC-MS spectrum of benzylamine (**5**) substrate.

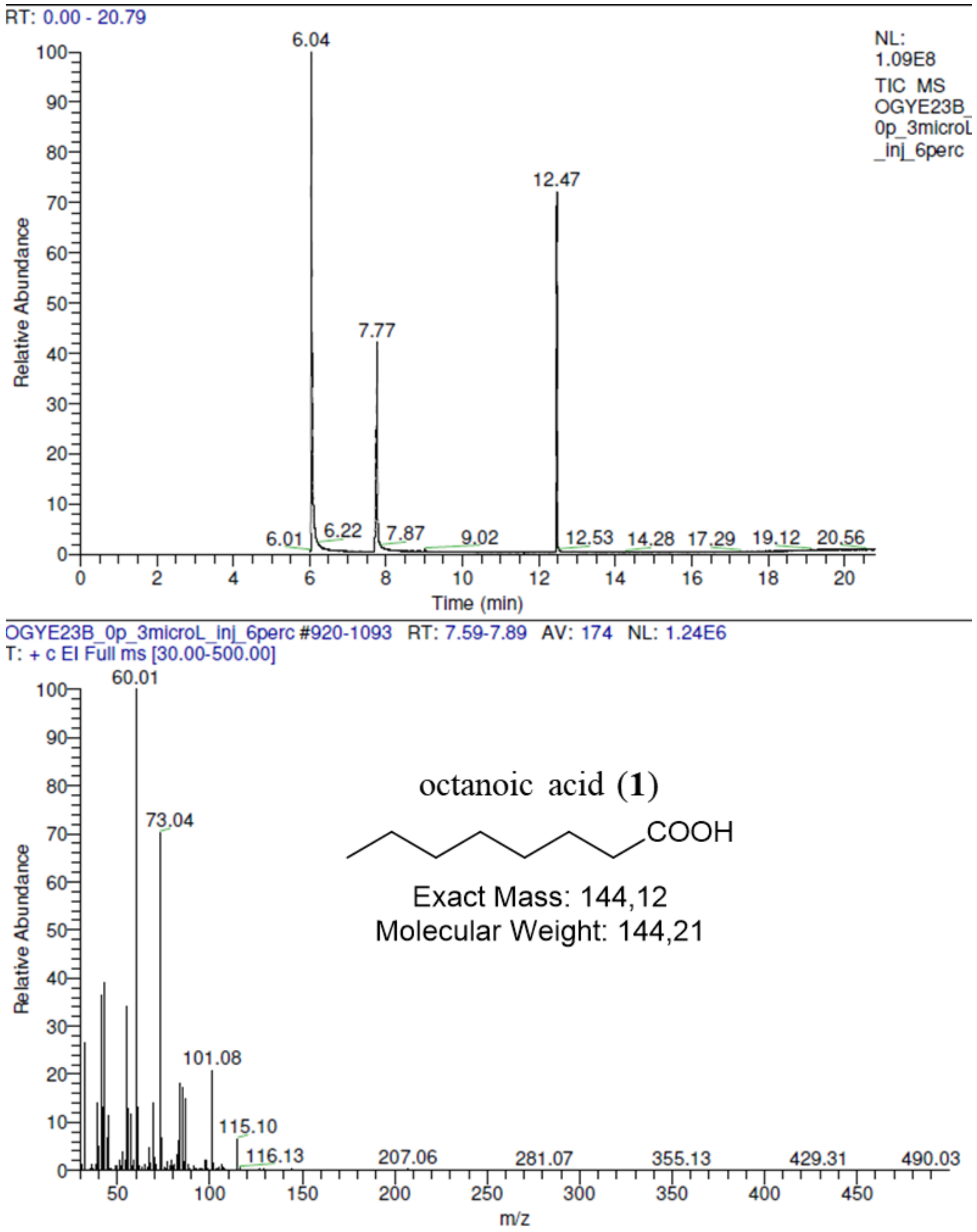

Figure S61. GC-MS spectrum of octanoic acid (**1**) substrate.

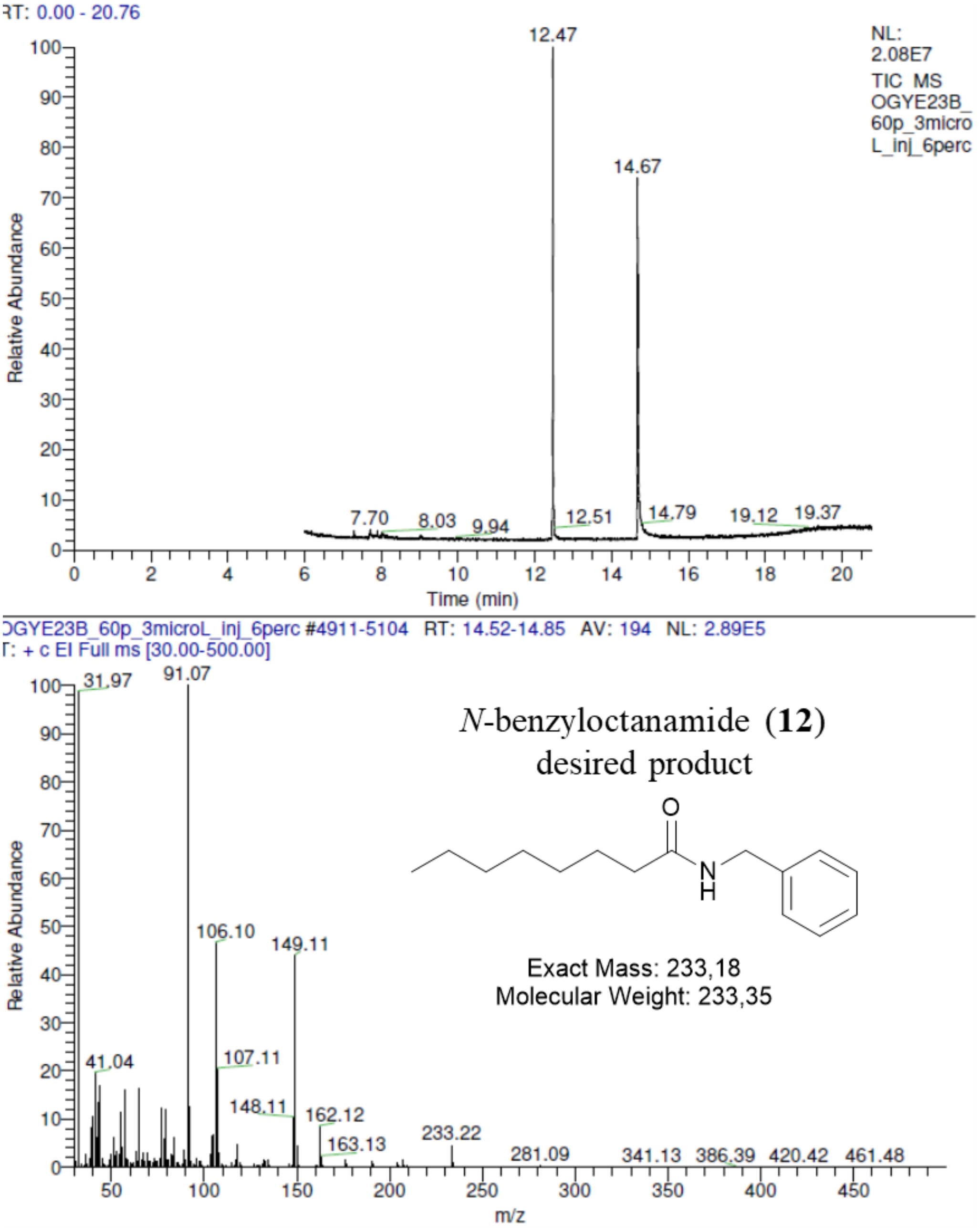

Figure S62. GC-MS spectrum of *N*-benzyloctanamide (**12**) product.
